# HBx represses WDR77 to enhance HBV replication by DDB1-mediated WDR77 degradation in the liver

**DOI:** 10.7150/thno.57531

**Published:** 2021-07-25

**Authors:** Hongfeng Yuan, Lina Zhao, Ying Yuan, Haolin Yun, Wei Zheng, Yu Geng, Guang Yang, Yufei Wang, Man Zhao, Xiaodong Zhang

**Affiliations:** 1 Department of Gastrointestinal Cancer Biology, Tianjin Cancer Institute, Liver Cancer Center, Tianjin Medical University Cancer Institute and Hospital, National Clinical Research Center for Cancer, Tianjin, P.R. China.; 2 Department of Cancer Research, College of Life Sciences, Nankai University, Tianjin, P.R. China.

**Keywords:** HBx, H4R3me2s, WDR77, PRMT5, DDB1

## Abstract

**Rationale:** Hepatitis B x protein (HBx) is required to initiate and maintain the replication of hepatitis B virus (HBV). Protein arginine methyltransferases 5 (PRMT5) negatively regulates HBV transcription. WD repeat domain 77 protein (WDR77) greatly enhances the methyltransferase activity of PRMT5. However, the role of WDR77 in the modulation of cccDNA transcription and HBV replication is poorly understood. In this study, we investigated the mechanism by which HBx modulated HBV replication involving WDR77 in the liver.

**Methods:** A human liver-chimeric mouse model was established. Immunohistochemistry (IHC) staining, Western blot analysis, Southern blot analysis, Northern blot analysis, immunofluorescence assays, ELISA, RT-qPCR, CoIP assays, and ChIP assays were performed in human liver-chimeric mouse model, primary human hepatocytes (PHHs), HepG2-NTCP, dHepaRG and HepG2 cell lines.

**Results:** HBV infection and HBx expression remarkably reduced the protein levels of WDR77 in human liver-chimeric mice and HepG2-NTCP cells. WDR77 restricted cccDNA transcription and HBV replication in PHHs and HepG2-NTCP cells. Mechanically, WDR77 enhanced PRMT5-triggered symmetric dimethylation of arginine 3 on H4 (H4R3me2s) on the cccDNA minichromosome to control cccDNA transcription. HBx drove the cellular DDB1-containing E3 ubiquitin ligase to degrade WDR77 through recruiting WDR77, leading to the disability of methyltransferase activity of PRMT5. Thus, HBx promoted HBV replication by driving a positive feedback loop of HBx-DDB1/WDR77/PRMT5/H4R3me2s/cccDNA/HBV/HBx in the liver.

**Conclusions:** HBx attenuates the WDR77-mediated HBV repression by driving DDB1-induced WDR77 degradation in the liver. Our finding provides new insights into the mechanism by which HBx enhances HBV replication in the liver.

## Introduction

Hepatitis B virus (HBV) infection is a major global public health issue that causes chronic hepatitis B (CHB), putting people at high risk for the progression of liver injury, such as liver failure, cirrhosis, and eventually hepatocellular carcinoma (HCC) 1, 2. HBV is a member of hepadnaviridae family containing a partially double-stranded circular DNA genome around 3.2 kb 3. After entry into the host cells, relaxed circular DNA (RC-DNA) is delivered into the nucleus and converted into the covalently closed circular DNA (cccDNA), which then serves as a template for all HBV viral RNAs. The mature nucleocapsids with HBV RC-DNA are either released from the cell after packaging with envelope proteins or recycled to the cccDNA reservoir in the nucleus. The cccDNA is crucial for viral persistence, and the currently antiviral drugs successfully suppress viral replication but fail to eradicate the cccDNA reservoir 4. HBV cccDNA organizes into a minichromosome with histone and nonhistone proteins, such as HBV core protein (HBc), HBV X protein (HBx), and host transcription factors 5. The level of HBV replication is determined by the number of cccDNA molecules and its transcriptional productivity 6. HBV cccDNA transcription activity is regulated by the host epigenetic mechanisms, including DNA methylation, histone modifications, and nucleosome remodeling 7, 8. A growing body of evidence suggests that post-translational modifications of histone on cccDNA minichromosome regulate the viral transcription. Histone acetylation and methylation (AcH3, H3K4me3, H3K9me3, H3K27me3, and H4R3me2s) and chromatin modification enzymes (PCAF/GCN5, p300/CBP, HDAC1, SIRT1, PRMT1, PRMT5, and EZH2) have been reported to be associated with the cccDNA transcriptional activity 9, 10. However, the mechanism by which the interaction between virus and host factors confers the modification of cccDNA minichromosome is not well documented.

HBx is a multi-functional protein of HBV, which trans-activates various cellular genes and pathways 3, 11. There is an abundance of data indicating that HBx is required to initiate and maintain HBV replication, likely through both direct and indirect mechanisms 12, 13. Viruses have limited genetic information and frequently usurp host cellular pathways and interact with host cellular proteins to facilitate their replication 14. HBx interacts with numerous host proteins and may mediate its biological functions through pleiotropic protein-protein interactions (PPIs) 15-17. Damage-specific DNA-binding protein 1 (DDB1) is a well-characterized HBx-binding partner, and the binding of HBx to DDB1 is critical for HBV replication 18. HBx activates cccDNA transcription by hijacking the cellular DDB1-containing E3 ligase to degrade HBV restriction factor the structural maintenance of chromosomes (SMC) complex SMC5/6 19, 20. Despite extensive studies on HBx-DDB1, the substrates and functional significance of HBx-DDB1 on HBV replication need to be further identified. However, the mechanism by which HBx modulates the cccDNA transcription is poorly understood.

Arginine methylation is a common post-translational modification of proteins, which plays a vital role in a variety of biological processes 21-23. Arginine methylation is catalyzed by protein arginine methyltransferases (PRMTs), including type I enzymes (PRMT1, 2, 3, 4, 6, and 8) which catalyze the production of monomethyl arginine (MMA) and asymmetric dimethylarginine (aDMA), type II PRMTs (PRMT5 and 9) which generate the MMA and symmetric dimethylarginine (sDMA), and type III enzyme (PRMT7) which catalyzes the formation of MMA 24. Interestingly, PRMT5 is the primary and well-described type II PRMTs, which is usually found in a complex containing WDR77 that greatly enhances PRMT5's methyltransferase activity through increasing the affinity for the protein substrate 25. PRMT5 can methylate H2A and H4 efficiently and H3 weakly, and H4R3me2s is a key histone methylation mark deposited by PRMT5, in which WDR77 as a non-catalytic component of the PRMT5-WDR77 complex, plays an essential role in the event 25. It has been reported that PRMT5 is a restrictor of HBV replication that triggers H4R3me2s on cccDNA through its methyltransferase activity to epigenetically block the cccDNA transcription 26. However, the effect of HBx on H4R3me2s in modulation of cccDNA minichromosome is elusive.

In this study, we try to identify the mechanism by which HBx epigenetically regulates cccDNA minichromosome in the liver. Interestingly, we found that HBx significantly reduced the methyltransferase activity of PRMT5 *in vitro* and *in vivo*, leading to the increase of cccDNA transcription. Moreover, we identified that WDR77 was a novel restrictor of HBV replication. HBx triggered the cellular DDB1-containing E3 ubiquitin ligase to degrade WDR77 through recruiting WDR77, leading to the disability of methyltransferase activity of PRMT5. Our finding provides new insights into the mechanism by which HBx enhances HBV replication.

## Results

### HBV infection leads to the decrease of PRMT5 methylase activity and WDR77 level

**Figure 1:**
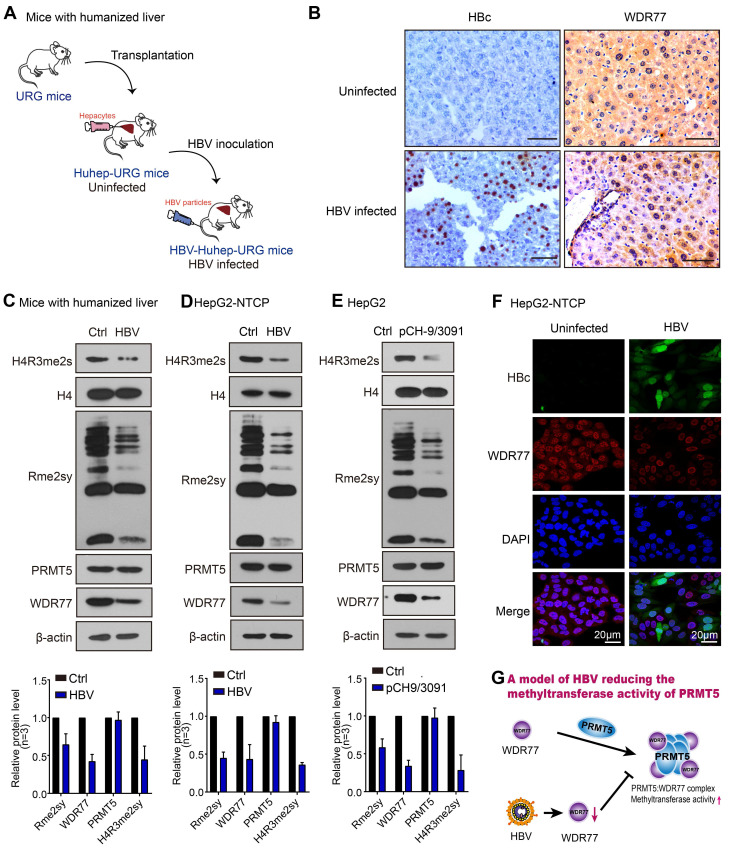
** HBV infection leads to the decrease of PRMT5 methylase activity and WDR77 level.** (A) A model of establishment of human liver-chimeric mice. (B) Immunohistochemistry assays for HBcAg and WDR77 were performed in the liver tissues from human liver-chimeric mice. N = 3 in each group. Scale bars: 50 μm. (C) The levels of H4R3me2s, Rme2sy, PRMT5, and WDR77 were examined by Western blot analysis in the liver tissues from human liver-chimeric mice. The quantification of the Western blot analysis for 3 experiments was shown (down). The other two Western tests were shown in Figure S1B. (D) HepG2-NTCP cells were uninfected or infected with wild-type HBV (at a multiplicity of infection of 1000 vp/cell). The levels of H4R3me2s, Rme2sy, PRMT5, and WDR77 were tested by Western blot analysis 7 days later. The quantification of the Western blot analysis for 3 experiments was shown (down). The other two Western tests were shown in Figure S1C. (E) HepG2 cells were transfected with pCH9 (vector control, 2 μg) or pCH9/3091 (HBV-expressing plasmid, 2 μg) plasmids. The levels of H4R3me2s, Rme2sy, PRMT5, and WDR77 were evaluated by Western blot analysis 3 days later. The quantification of Western blot analysis for 3 experiments was shown (down). The other two Western tests were shown in Figure S1D. (F) HepG2-NTCP cells were uninfected or infected with wild-type HBV (at a multiplicity of infection of 1000 vp/cell). The expression of HBcAg and WDR77 was assessed by immunofluorescence assays 7 days later. Scale bars: 10 μm. (G) A model of HBV reducing the methyltransferase activity of PRMT5.

Given that PRMT5 catalyzed H4R3me2s negatively regulates HBV replication 26, we wondered whether HBV abolished PRMT5 methylase activity to release the inhibition of HBV replication. To answer this question, we established the human-liver chimeric mouse model (Huhep-URG mice model) to evaluate the effect of HBV infection on PRMT5 methylase activity *in vivo* (Figure 1A; Table S1). Immunohistochemistry (IHC) staining revealed that the levels of H4R3me2s and WDR77 were significantly decreased in the liver tissues from the HBV-infected human-liver chimeric mice (Figure 1B; Figure S1A). IHC staining displayed that human albumin (hALB) was positive in the liver tissues of Huhep-URG mice (Figure S1A), and the percentage of humanization of the livers was about 50%. Western blot analysis showed that the global levels of symmetrical methylation of arginine residues (Rme2sy) and H4R3me2s were markedly decreased in the liver tissues (Figure 1C; Figure S1B), suggesting that HBV infection reduces the methylase activity of PRMT5 *in vivo*. Furthermore, our results showed that HBV *de novo* infection (or transfected with HBV-expressing pCH-9/3091 plasmid) obviously reduced the levels of Rme2sy and H4R3me2s in HepG2-NTCP and HepG2 cells (Figure 1D-E; Table S2; Figure S1C-D), suggesting that HBV replication attenuates the methylase activity of PRMT5 *in vitro*.

WDR77 is the core component of the PRMT5 multimeric complex, which is required for the interaction of PRMT5 with binding partners and substrates of PRMT5 to enhance its methylase activity 27. Surprisingly, IHC staining showed that HBV infection reduced the levels of WDR77, but not PRMT5, in the liver tissues from the HBV-infected human-liver chimeric mice (Figure 1B; Figure S1A). Western blot analysis confirmed that HBV replication reduced the protein levels of WDR77, but not PRMT5 (Figure 1C-E; Figure S1B-D), implying that WDR77 is involved in the event of HBV reducing the methylase activity of PRMT5. Meanwhile, immunofluorescence staining revealed that HBV infected HepG2-NTCP cells expressed significantly lower levels of WDR77 (Figure 1F). Thus, we conclude that HBV infection results in the decrease of PRMT5 methylase activity and WDR77 level, which is summarized in a model (Figure 1G).

### HBx decreases the PRMT5 methylase activity and WDR77 level

**Figure 2:**
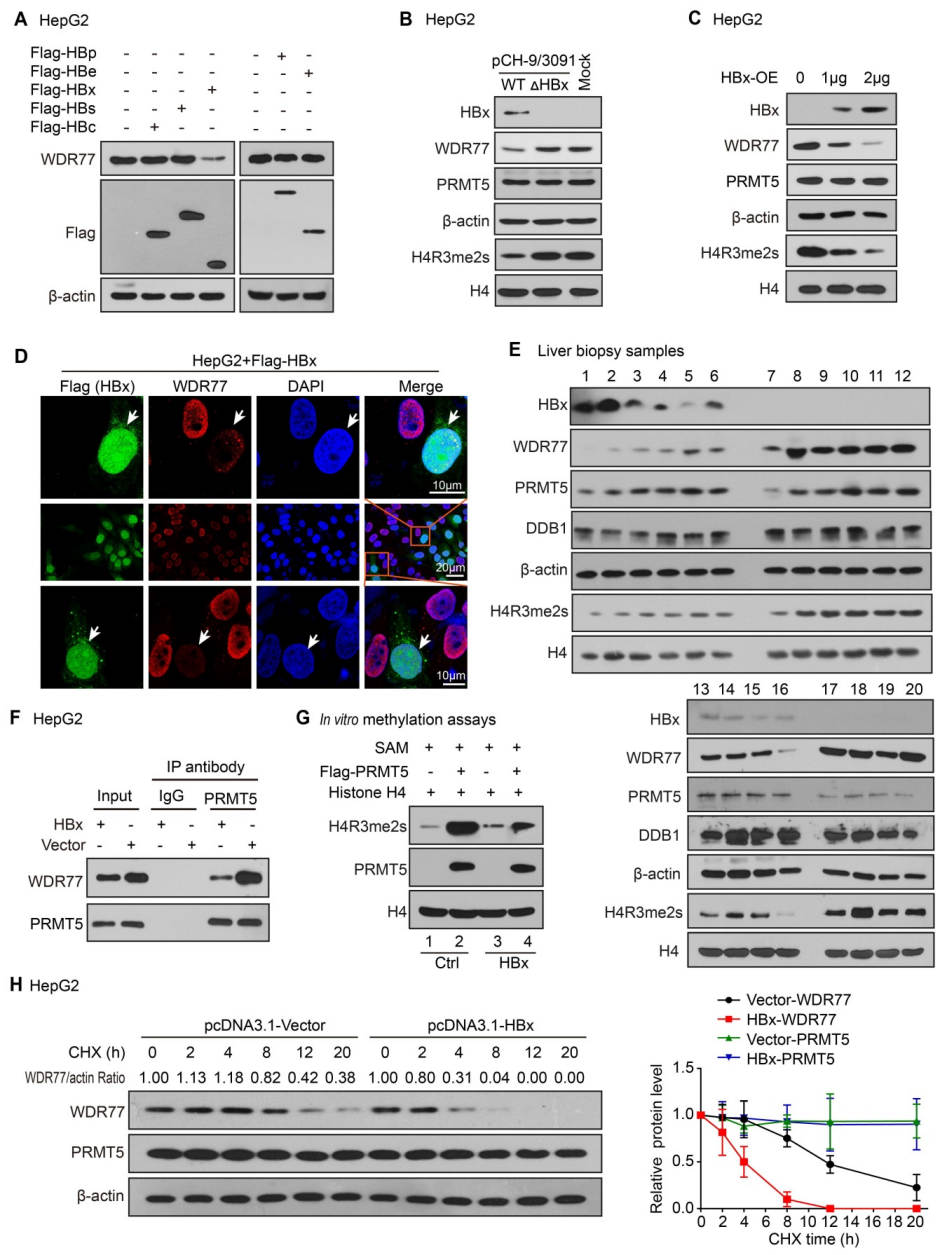
** HBx decreases the PRMT5 methylase activity and WDR77 level.** (A) HepG2 cells were transfected with Flag-tagged HBp, HBe, HBx, HBs, and HBc plasmids (2 μg), respectively. The effect of HBp, HBe, HBx, HBs, and HBc on WDR77 was detected by Western blot analysis 3 days later. (B) HepG2 cells were transfected with pCH-9/3091 (WT, 2 μg) or HBx-deficient pCH-9/3091(ΔHBx, 2 μg) plasmids. The levels of HBx, WDR77, PRMT5, and H4R3me2s were tested by Western blot analysis 3 days later. (C) HepG2 cells were transfected with pcDNA3.1-HBx (2 μg) or pcDNA3.1-Vector plasmids (2 μg). The levels of HBx, WDR77, PRMT5, and H4R3me2s were measured by Western blot analysis 3 days later. (D) HepG2 cells were transfected with Flag-tagged HBx plasmids (2 μg). The expression of WDR77 (red) and HBx (green) was assessed by immunofluorescence assays 3 days later. (E) The levels of HBx, WDR77, PRMT5, DDB1 and H4R3me2s were examined by Western blot analysis in the clinical needle biopsy liver tissues. (F) HepG2 cells were transfected with pcDNA3.1-HBx (2 μg) or pcDNA3.1-Vector (2 μg) plasmids. PRMT5 was immunoprecipitated by anti-PRMT5 antibody from the cells, and the levels of WDR77 were analyzed by CoIP analysis 3 days later. (G) HepG2-WDR77 cells were co-transfected with Flag-PRMT5 and pcDNA3.1-HBx (2 μg) or pcDNA3.1-Vector (2 μg) plasmids. An *in vitro* methylation assays were performed by using Flag-PRMT5 purified from the cells 3 days later. (H) HepG2 cells were transfected with pcDNA3.1-HBx or pcDNA3.1-Vector 48 h followed by cycloheximide (CHX) (100 μg/mL) treatment for the indicated time. The protein levels of WDR77 and PRMT5 were detected by Western blot analysis. The quantitative analysis of WDR77 and PRMT5 expression was shown (right).

Next, we try to identify which component of HBV influencing the levels of WDR77 in the liver. Based on that HBV genome contains four open reading frames encoding seven proteins, including the viral DNA polymerase (P), three viral envelope proteins (PreS1, PreS2 or S), core proteins (PreC or C) and HBx 28, 29, we overexpressed Flag-tagged HBx, HBs, HBc, HBp and HBe in HepG2 cells, and tested the changes of WDR77 expression in the cells. Western blot analysis and RT-qPCR revealed that HBx reduced the protein levels of WDR77, but not the mRNA levels of WDR77, in the cells (Figure 2A; Figure S2A-B). Our data showed that absence of HBx rescued WDR77 levels in the HepG2 cells transfected with pCH-9/3091 mutant plasmid (ΔHBx, HBx-deficient HBV plasmid), relative to transfected with pCH-9/3091 plasmid (WT, wild-type HBV plasmid) (Figure 2B; Figure S2C). Moreover, we observed that HBx reduced the levels of WDR77 and H4R3me2s, but not PRMT5, in HepG2 cells transfected with pcDNA3.1-HBx plasmid (Figure 2C; Figure S2D), implying that WDR77 is involved in the event of HBx reducing the methylase activity of PRMT5. Immunofluorescence analysis confirmed the effects of HBx on WDR77 expression in HepG2 and HEK293T cells transfected with HBx plasmid (Figure 2D; Figure S2E). Clinically, Western blot analysis validated that the protein levels of WDR77 and H4R3me2s were lower in the HBx-positive liver biopsy samples from hepatitis B patients (Figure 2E). Coimmunoprecipitation (CoIP) assays showed that the binding of WDR77 to PRMT5 was reduced in HBx-overexpressing HepG2 cell lines (Figure 2F; Figure S2F). We purified Flag-tagged PRMT5 protein from HepG2-WDR77 cells co-transfected with PRMT5 with HBx or vector plasmids, and then incubated the purified proteins with SAM and recombinant histone H4 in an *in vitro* methylation assay. As expected, co-expression of HBx with PRMT5 greatly impaired the methylase activity of PRMT5 (Figure 2G; Figure S2G). Cycloheximide (CHX) chase experiment showed that HBx reduced the half-life of WDR77 protein (Figure 2H; Figure S2H), suggesting that HBx leads to the instability of WDR77 in the cells. Collectively, we conclude that HBx confers the decrease of PRMT5 methylase activity and WDR77 level.

### WDR77 represses the HBV replication

**Figure 3:**
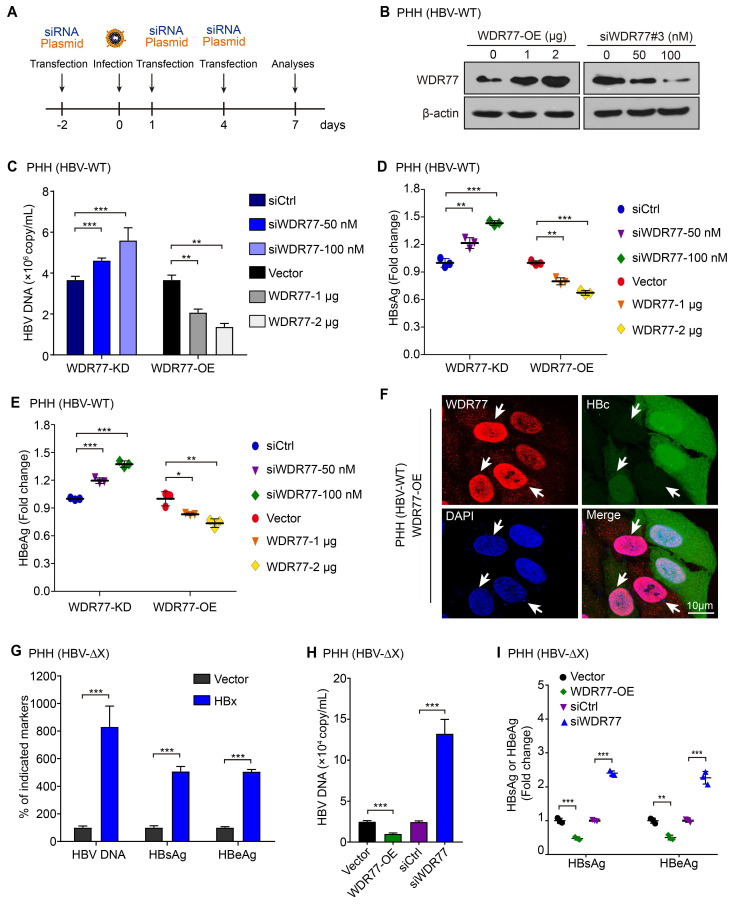
** WDR77 represses the HBV replication.** (A) Schematic representation of cell experimental process. (B) PHH cells were infected with wild-type HBV (at a multiplicity of infection of 1000 vp/cell) and were continuously transfected with indicated plasmids or siWDR77 (siWDR77#3) at -2, 1, and 4 dpi (days post-infection). The levels of WDR77 were assessed by Western blot analysis 7 days post-infection. (C) The levels of HBV DNA were measured by qPCR in the supernatant of HBV *de novo* infection PHH cells transfected with siWDR77 or pcDNA3.1-WDR77. (D) The levels of HBsAg were examined by ELISA in the supernatant of HBV *de novo* infection PHH cells transfected with siWDR77 or pcDNA3.1-WDR77. (E) The levels of HBeAg were determined by ELISA in the supernatant of HBV *de novo* infection PHH cells transfected with siWDR77 or pcDNA3.1-WDR77. (F) The expression of HBc (green) and WDR77 (red) was determined by immunofluorescence staining in HBV *de novo* infection PHH cells transfected with pcDNA3.1-WDR77 (2 μg). Scale bars, 10 μm. (G) PHH cells were infected with HBx-deficient HBV (ΔX, at a multiplicity of infection of 1000 vp/cell) and were continuously transfected with indicated plasmids or siRNA of WDR77 at -2, 1, and 4 dpi (days post-infection). The levels of HBV DNA, HBsAg and HBeAg were measured by qPCR and ELISA in the supernatant of the cells 7 days post-infection. (H) The levels of HBV DNA were determined by qPCR in the supernatant of HBx-deficient HBV *de novo* infection PHH cells (ΔX) transfected with siCtrl (100 nM), siWDR77 (100 nM), pcDNA3.1-Vector (2 μg), or pcDNA3.1-WDR77 (WDR77-OE, 2 μg). (I) The levels of HBsAg and HBeAg were examined by ELISA in the supernatant of HBx-deficient HBV *de novo* infection PHH cells (ΔX) transfected with siCtrl (100 nM), siWDR77 (100 nM), pcDNA3.1-Vector (2 μg), or pcDNA3.1-WDR77 (WDR77-OE, 2 μg). Data are represented as means ± SD (n = 3). Student's *t* test, *p < 0.05; **p < 0.01; ***p < 0.001.

Given that PRMT5 plays a vital role in the restriction of HBV transcription during HBV infection 26, we supposed that WDR77 might be involved in the methylase activity of PRMT5 that repressed the cccDNA transcription. The efficiencies of WDR77 siRNA were evaluated by RT-qPCR and Western blot analysis in HepG2 cells (Figure S3A), and siWDR77#3 (termed siWDR77 or WDR77-KD) was selected for subsequent experiments. The experiments to evaluate the effect of WDR77 on HBV replication were designed in a model (Figure 3A). To screen the effective dose of HBV infection, we measured the efficiency of HBV infection in a different multiplicity of infection (MOI) of viral particles (vp) in PHH, and found that over 500 vp/cell was available in the system (Figure S3B-D). Real time PCR, ELISA and immunofluorescence analysis showed that WDR77-KD elevated the levels of HBV DNA, HBsAg, HBeAg, and HBc in HBV *de novo* infection PHH cells (HBV-WT) in a dose-dependent manner (Figure 3B-F). Conversely, WDR77-OE led to significant down-regulation of HBV DNA, HBsAg, HBeAg, and HBc in the cells in a dose-dependent manner (Figure 3B-F). Mutant HBV lacking a functional HBx gene (HBV-ΔX) was fully competent to enter cells in our system (Figure S3B). We performed HBx rescued assay and found that HBx-OE could elevate the HBV markers (Figure 3G). Meanwhile, we confirmed the antiviral effects of WDR77 in the HBV-ΔX infected PHH cells (Figure 3H-I), suggesting that WDR77 is a restrictor of HBV replication. Thus, we conclude that WDR77 represses the HBV replication in the cells.

### WDR77 attenuates the transcription activity of cccDNA

**Figure 4:**
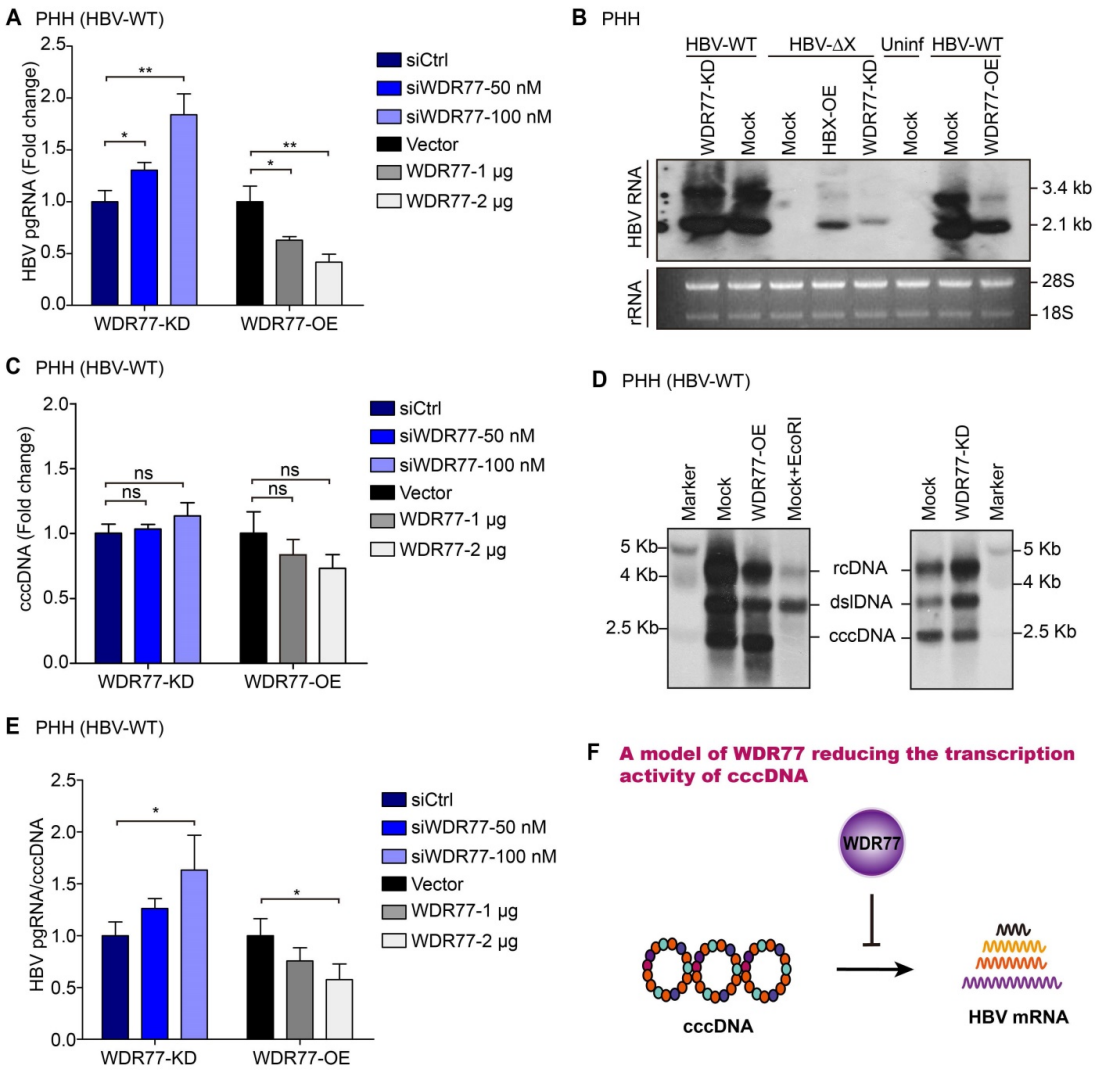
** WDR77 attenuates the transcription activity of cccDNA.** (A) PHH cells were infected with wild-type HBV (at a multiplicity of infection of 1000 vp/cell) and were continuously transfected with plasmids or siRNA of WDR77 at -2, 1, and 4 dpi (days post-infection). The levels of HBV pgRNA were quantified by RT-qPCR assays 7 days post-infection. (B) PHH cells were infected with wild-type or HBx-deficient HBV (at a multiplicity of infection of 1000 vp/cell) and were continuously transfected with pcDNA3.1-HBx (10 μg), siWDR77 (100 nM) or pcDNA3.1-WDR77 (10 μg) at -2, 1, and 4 dpi (days post-infection). The levels of HBV RNA were tested by Northern blot analysis 7 days post-infection. (C) PHH cells were infected with wild-type HBV (at a multiplicity of infection of 1000 vp/cell) and were continuously transfected with siWDR77 or pcDNA3.1-WDR77 at -2, 1, and 4 dpi (days post-infection). The levels of cccDNA were evaluated by RT-qPCR analysis 7 days post-infection. (D) PHH cells were infected with wild-type HBV (at a multiplicity of infection of 1000 vp/cell) and were continuously transfected with siWDR77 (100 nM) or pcDNA3.1-WDR77 (10 μg) at -2, 1, and 4 dpi (days post-infection). The levels of cccDNA were tested by Southern blot analysis 7 days post-infection. (E) HBV transcription activity was assessed by calculating the ratio of pgRNA/cccDNA. (F) A model of WDR77 reducing the transcription activity of cccDNA. Data are represented as means ± SD (n = 3). Student's *t* test, ns, no significant; *p < 0.05; **p < 0.01; ***p < 0.001.

Next, we examined the effect of WDR77 on the levels of HBV mRNA. Consistently, RT-qPCR assays showed that the knockdown or overexpression of WDR77 significantly increased or decreased the levels of HBV pgRNA in HBV *de novo* infection PHH and HepG2-NTCP cells, respectively (Figure 4A, Figure S4A). We performed Northern blot analysis to confirm the conclusion. However, the viral gene transcription was undetectable in PHH infected with mutant HBV lacking a functional HBx gene (HBV-ΔX) (Figure 4B; Figure S4B). Our data showed that overexpression of HBx (HBx-OE) and depletion of WDR77 (WDR77-KD) could restore viral gene transcription competency in PHH cells infected with HBx-deficient virus (Figure 4B; Figure S4B). The knockdown (or overexpression) of WDR77 could slightly increase (or decrease) the replication of the wild-type virus (Figure 4B; Figure S4B). Real-time PCR and Southern blot analysis showed that WDR77 had no significant effect on the levels of cccDNA (Figure 4C-D; Figure S4C). Accordingly, the ratio of pgRNA to cccDNA presented the transcriptional activity of HBV cccDNA 26. As expected, WDR77 significantly regulated the ratio of pgRNA to cccDNA in HBV *de novo* infection PHH and HepG2-NTCP cells (Figure 4E, Figure S4D). Overall, we conclude that WDR77 is capable of inhibiting the transcription activity of cccDNA in a model (Figure 4F).

### WDR77 is required for the PRMT5-mediated inhibition of cccDNA transcription

**Figure 5:**
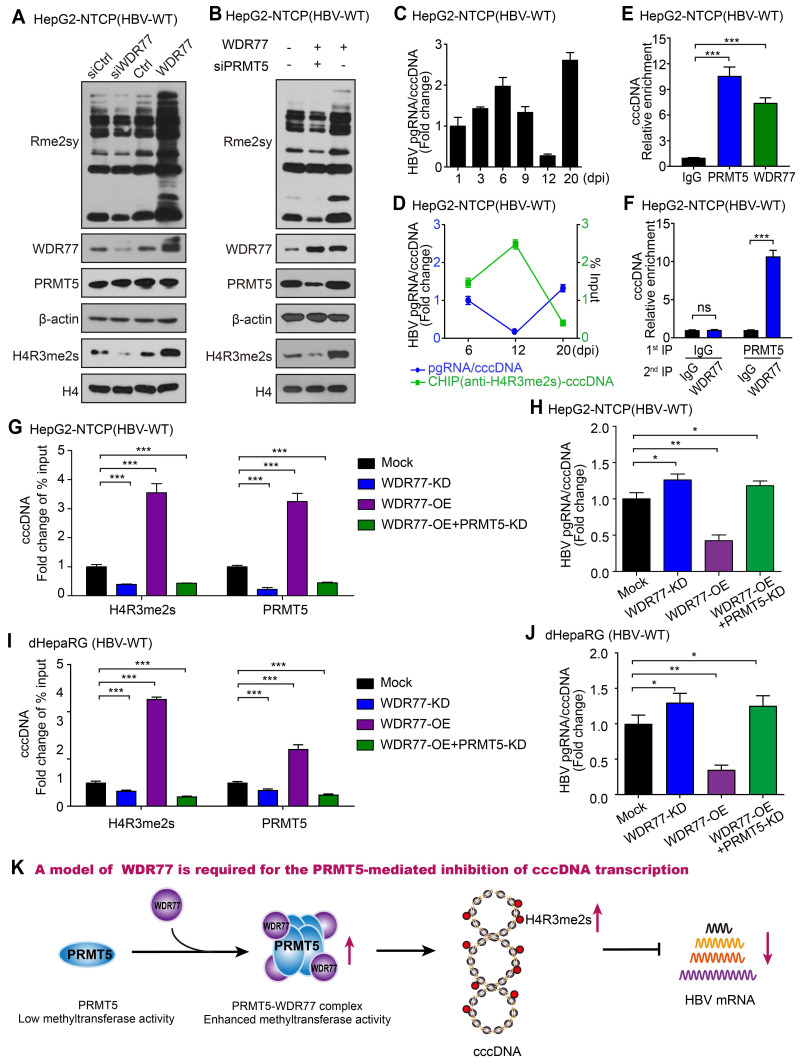
** WDR77 is required for the PRMT5-mediated inhibition of cccDNA transcription.** (A) HepG2-NTCP cells were infected with wild-type HBV (at a multiplicity of infection of 1000 vp/cell) and were continuously transfected with siWDR77 (100 nM) or pcDNA3.1-WDR77 (2 μg) at -2, 1, and 4 dpi (days post-infection). The levels of Rme2sy, WDR77, PRMT5 and H4R3me2s were detected by Western blot analysis 7 days post-infection. (B) HepG2-NTCP cells were infected with wild-type HBV (at a multiplicity of infection of 1000 vp/cell) and were continuously transfected with siPRMT5 (100 nM) or pcDNA3.1-WDR77 (2 μg) at -2, 1, and 4 dpi (days post-infection). The levels of Rme2sy, WDR77, PRMT5 and H4R3me2s were evaluated by Western blot analysis 7 days post-infection. (C) HBV transcription activity was assessed by calculating the ratio of pgRNA/cccDNA in HBV *de novo* infection HepG2-NTCP cells at the indicated days after HBV infection. (D) The assembly of H4R3me2s onto cccDNA was examined by ChIP-qPCR in HBV *de novo* infection HepG2-NTCP cells in the indicated days after HBV infection. (E) HepG2-NTCP cells were infected with wild-type HBV (at a multiplicity of infection of 1000 vp/cell). The assembly of PRMT5 or WDR77 onto cccDNA was determined by ChIP-qPCR 7 days later. (F) HepG2-NTCP cells were infected with wild-type HBV (at a multiplicity of infection of 1000 vp/cell). Re-ChIP assay was performed with the indicated antibodies 7 days later. (G) The assembly of H4R3me2s onto cccDNA was determined by ChIP-qPCR in HBV *de novo* infection HepG2-NTCP cells transfected with siWDR77 (100 nM), pcDNA3.1-WDR77 (10 μg) or co-transfected with pcDNA3.1-WDR77 (10 μg) and siPRMT5 (100 nM). (H) HBV transcription activity was evaluated by calculating the ratio of pgRNA/cccDNA in HBV *de novo* infection HepG2-NTCP cells transfected with siWDR77 (100 nM), pcDNA3.1-WDR77 (10 μg) or co-transfected with pcDNA3.1-WDR77 (100 nM) and siPRMT5 (10 μg). (I) The assembly of H4R3me2s onto cccDNA was determined by ChIP-qPCR in HBV *de novo* infection dHepaRG cells transfected with siWDR77 (100 nM), pcDNA3.1-WDR77 (10 μg) or co-transfected with pcDNA3.1-WDR77 (10 μg) and siPRMT5 (100 nM). (J) HBV transcription activity was evaluated by calculating the ratio of pgRNA/cccDNA in HBV *de novo* infection dHepaRG cells transfected with siWDR77 (100 nM), pcDNA3.1-WDR77 (10 μg) or co-transfected with pcDNA3.1-WDR77 (10 μg) and siPRMT5 (100 nM). (K) A model of WDR77 suppressing cccDNA transcription based on PRMT5 methyltransferase activity. Data are represented as means ± SD (n = 3). Student's *t* test, *p < 0.05; **p < 0.01; ***p < 0.001.

It has been reported that the PRMT5-WDR77 complex is a functional biological module, which has a higher methyltransferase activity compared with PRMT5 alone under similar experimental conditions 25. PRMT5-WDR77 complex methylates histones H2AR3, H3R2, H3R8, and H4R3 and affects transcriptional regulation 30, 31, and H4R3me2s is reported to bind to the HBV cccDNA minichromosome 26. Therefore, we speculated that the role of WDR77 against HBV might depend on the methyltransferase activity of PRMT5. Strikingly, Western blot analysis showed that WDR77 knockdown reduced the levels of Rme2sy and H4R3me2s in HBV *de novo* infection HepG2-NTCP cells (Figure 5A; Figure S5A). Conversely, WDR77 overexpression increased the levels of Rme2sy and H4R3me2s in the cells (Figure 5A; Figure S5A). The siPRMT5 (siPRMT5#3, termed as PRMT5-KD) attenuated the WDR77-mediated enhancement of Rme2sy and H4R3me2s in the cells (Figure 5B; Figure S5B-C). It has been reported that cccDNA transcriptional activity is negatively correlated with the levels of cccDNA-bound H4R3me2s 26. Consistently, ChIP analysis validated that the H4R3me2s recruitment on the cccDNA impaired the cccDNA transcriptional activity during HBV replication (Figure 5C-D, Figure S5D-F). ChIP analysis showed that both PRMT5 and WDR77 bound to cccDNA, and the Re-ChIP analysis indicated that PRMT5 and WDR77 localized to the same cccDNA genomic locus (Figure 5E-F, Figure S5G). Moreover, ChIP analysis showed that WDR77-OE (or WDR77-KD) led to the increase (or decrease) of both PRMT5 and H4R3me2s binding to cccDNA (Figure 5G, Figure S5H). Consistently, WDR77-OE (or WDR77-KD) resulted in the decrease (or increase) of cccDNA transcription activity in HBV *de novo* infection HepG2-NTCP cells (Figure 5H; Figure S5I-J). In addition, PRMT5-KD could block the effect of WDR77-OE on the status of H4R3me2s and the cccDNA transcriptional activity in the cells (Figure 5G-H). Meanwhile, we confirmed the effects of WDR77 in the HBV* de novo* infection dHepaRG cells (Figure 5I-J; Figure S5K-M), suggesting that WDR77 works depending on PRMT5. Together, we conclude that WDR77 is required for the PRMT5-mediated inhibition of cccDNA transcription in a model (Figure 5K).

### HBx degrades WDR77 by DDB1-CUL4-ROC1 E3 ligase

**Figure 6:**
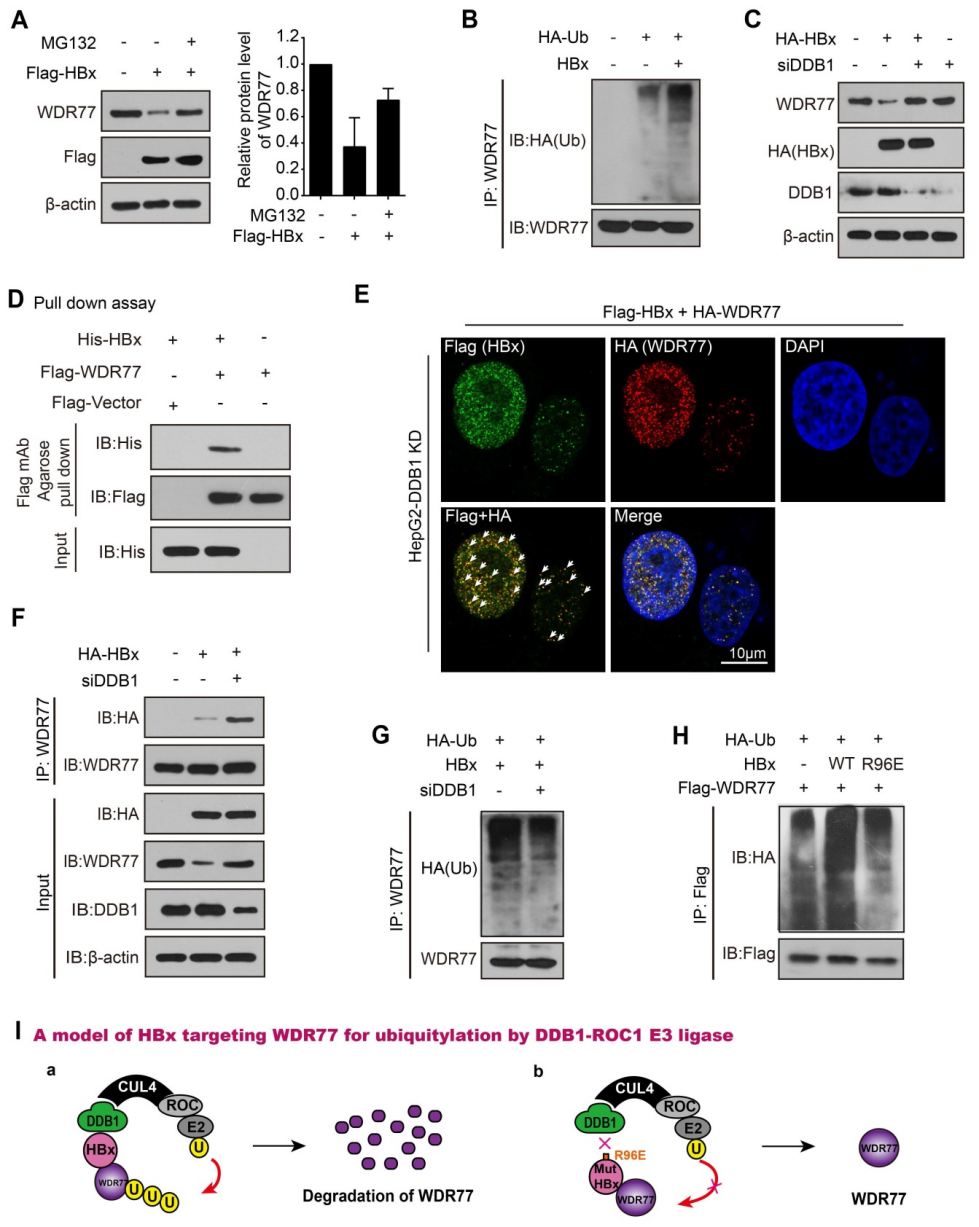
** HBx degrades WDR77 by DDB1-CUL4-ROC1 E3 ligase.** (A) HepG2 cells were transfected with Flag-tagged HBx (2 μg) for 48 h and then treated with MG132 (2 mM) for 24 h. The protein levels of WDR77 were detected by Western blot analysis in the cells. (B) HepG2 cells were transfected with the indicated plasmids (pcDNA3.1-HBx and HA-tagged Ubiquitin, 10 μg) and treated with MG132 (2 mM) 4 h before harvest. The ubiquitylation (Ub) of immunoprecipitated WDR77 was examined by Western blot analysis in the cells 3 days later. (C) HepG2 cells were transfected with HA-tagged HBx (2 μg) and/or siDDB1 (100 nM). The protein levels of WDR77, HBx and DDB1 were measured by Western blot analysis in the cells 3 days later. (D) His-tagged HBx was purified from E.coli with Ni-NTA resin. Flag-tagged WDR77 was purified by anti-DDDDK-tag mAb-magnetic agarose from HEK293T cells transfected with Flag-tagged WDR77 (10 μg). The beads with Flag-WDR77 were incubated with His-HBx and examined by immunoblotting with anti-His antibody. (E) HepG2 cells were co-transfected with Flag-tagged HBx (2 μg), HA-tagged WDR77 (2 μg), and siDDB1 (100 nM). The co-localization of exogenous Flag-tagged HBx (green) and HA-tagged WDR77 (red) were determined by immunofluorescence staining with confocal microscopy 3 days later. Scale bars, 10 μm. (F) HepG2 cells were transfected with HA-tagged HBx (10 μg) and/or siDDB1 (100 nM). The binding of HBx to immunoprecipitated WDR77 was examined by Western blot analysis 3 days later. (G) HepG2 cells were co-transfected with pcDNA3.1-HBx (10 μg), HA-tagged Ubiquitin (10 μg) and siDDB1 (100 nM), followed by treatment with MG132 (2 mM) 4 h before harvest. Ubiquitylation of immunoprecipitated WDR77 was tested by Western blot analysis 3 days later. (H) HepG2 cells were co-transfected with pcDNA3.1-HBx/pcDNA3.1-HBx mut (10 μg), HA-tagged Ubiquitin (10 μg) and Flag-tagged WDR77 (10 μg), followed by treatment with MG132 (2 mM) 4 h before harvest. Ubiquitylation of immunoprecipitated WDR77 was evaluated by Western blot analysis 3 days later. (I) A model of HBx targeting WDR77 for ubiquitylation by DDB1-ROC1 E3 ligase. a, HBx binds to DDB1 and recruits WDR77 for ubiquitylation by DDB1-ROC1 E3 ligase. b, HBx mutant failed to bind to DDB1 and DDB1-ROC1 E3 ligase cannot work.

We next examined the underlying mechanism by which HBx decreased the levels and stability of WDR77 protein. It has been reported that HBx is able to interact with DDB1-CUL4-ROC1 (CRL4) E3 ligase 19. Therefore, HBx might act either to recruit host restriction factors for ubiquitination and degradation, or to alter the CRL4 substrate specificity. Following the treatment with a proteasome inhibitor MG132, the CHX-induced WDR77 down-regulation could be reversed, and the polyubiquitylation signal of WDR77 was enhanced upon the addition of MG132 relative to control in the cells (Figure S6A), suggesting that WDR77 can be degraded in the ubiquitin-proteasome pathway. As expected, the co-treatment with MG132 and HBx largely blocked the effect of HBx on the accumulation of WDR77 in HepG2 cells (Figure 6A; Figure S6B), indicating that HBx may promote the proteasome-dependent degradation of WDR77 in the cells. In addition, the ubiquitination assays showed that HBx resulted in the substantial increase of WDR77 ubiquitination in HepG2 cells (Figure 6B; Figure S6C), suggesting that HBx promotes the ubiquitination of WDR77.

Previous studies suggested that HBx stimulates transcription of HBV genome by binding the DDB1 subunit of the DDB1-containing E3 ubiquitin ligase to target unknown host factors for ubiquitin-mediated degradation 32-34. Then, we examined whether HBx promoted the degradation of WDR77 by hijacking DDB1-containing E3 ubiquitin ligase. We designed four siRNAs against DDB1 and detected the efficiency of siRNAs by RT-qPCR and Western blot analysis, and siDDB1#4 (termed as PRMT5-KD or siDDB1) was the most effective siRNA among them and thus used for subsequent experiments (Figure S6D). Our data showed that DDB1 knockdown restored the WDR77 protein levels when HBx was overexpressed in HepG2 cells (Figure 6C; Figure S6E). Immunofluorescence assay presented the similar results in the cells (Figure S6F).

Interestingly, CoIP assays revealed that the interaction of WDR77 with HBx was undetectable in HBV *de novo* infection HepG2-NTCP cells that without any intervention to DDB1 (Figure S6G). However, pull-down assays (absence of DDB1) demonstrated that HBx was able to interact with WDR77 *in vitro* (Figure 6D; Figure S6H). Immunofluorescence colocalization was performed that exogenous Flag-tagged HBx and HA-tagged WDR77 were co-localized in HepG2 cells treated with siDDB1 (Figure 6E, Figure S6I), supporting that HBx interacts with WDR77. Real-time PCR and ELISA assay showed that DDB1 knockdown reduced the levels of HBV DNA, HBsAg and HBeAg in HBV *de novo* infection HepG2-NTCP cells (Figure S6J), suggesting that the depletion of DDB1 by siRNA inhibits the viral production and gene expression of HBV. Meanwhile, CoIP experiments validated that DDB1 knockdown increased the binding between HBx and WDR77 (Figure 6F; Figure S6K), implying that DDB1 knockdown could rescue the HBx-mediated degradation of WDR77 to restore the role of WDR77 in the repression of HBV. As expected, Western blot analysis validated that DDB1 knockdown reduced the polyubiquitylation of WDR77 in HepG2 cells transfected with HBx plasmids (Figure 6G; Figure S6L). HBx bound to DDB1 through an alpha-helical motif, and the interaction could be inhibited by HBx R96E point mutation 34. We generated a mutant of HBx with an “R96E”. Our data demonstrated that the mutant HBx (R96E) failed to induce the enhancement of polyubiquitylation of WDR77 relative to wild type HBx in the system (Figure 6H; Figure S6M), suggesting that HBx targets WDR77 for ubiquitylation by hijacking the cellular DDB1-containing E3 ligase. Taken together, we conclude that HBx degrades WDR77 by DDB1-CUL4-ROC1 E3 ligase in a model (Figure 6I).

## Discussion

HBV infection is a major global public health issue, in which cccDNA plays a crucial role in the viral persistence 35. The effect of host-virus interaction on the modification of cccDNA minichromosome has so far remained mostly unexplored. HBx enhances the cccDNA transcription 5. PRMT5 catalyzed H4R3me2s negatively regulates HBV replication 26. PRMT5-WDR77 complex is the functional biological module 25. However, the role of WDR77 in the modulation of cccDNA transcription and HBV replication is poorly understood. In this study, we identified the mechanism by which HBx epigenetically regulated the cccDNA minichromosome involving WDR77 in the liver.

Recently, the protein post-translational modifications (PTMs) on HBV cccDNA minichromosome, including acetylation, methylation, phosphorylation, ubiquitination, and so on, have become a hot point 9, 36. A growing number of epigenetic markers on the HBV cccDNA minichromosome that regulate the viral transcription have been identified, such as asymmetrical demethylation or symmetrical methylation on H4R3 (H4R3me2a and H4R3me2s) 9, 35, 37, 38. The chromatin modification enzymes that add and remove such modifications (p300/CBP, HAT1, and PRMT5) have been shown to be associated with cccDNA transcriptional repression or activation 7, 26. Notably, PRMT5-catalyzed H4R3me2s represses cccDNA transcription and plays a vital role in the restriction of HBV transcription during HBV infection 26. Accordingly, we were concerned whether HBV modulated PRMT5-catalyzed H4R3me2s in the liver. As expected, we validated that HBV could reduce the methylase activity of PRMT5, but not the levels of PRMT5 in hepatoma cells. Interestingly, we found that HBV down-regulated WDR77 in human liver-chimeric mice liver tissues, HBV plasmid transfected HepG2 cells and HBV *de novo* infection HepG2-NTCP cells. It suggests that HBV can reduce the methylase activity of PRMT5 and attenuate the protein levels of WDR77 in the cells. Consistently, our data confirmed with the previous study that viruses generally can inhibit host restrictors to promote viral gene expression following infection 39. It has been reported that Smc5/6 complex is identified as a host restriction factor, and HBx promotes the degradation of SMC5/6 to enhance HBV replication 19, 20. Based on that, we identified that HBx was responsible for the events. Our results showed that HBV would trigger strong host genome and cccDNA epigenetic changes such as H4R3me2s. It has been reported that HBV infection affects the levels of acetylation of H3K27, H4K5 and H4K12 in the liver of human liver-chimeric mice 7. HBV is capable of inducing a genome-wide change in H3K4me3 and H3K27me3 modifications in HepG2 cells 40. HBx co-localizes with WDR5 on chromatin genome-wide and promotes genome-wide H3K4me3 modification via WDR5 41. Our data are consistent with these reports.

PRMT5 plays a vital role in the restriction of HBV transcription during HBV infection 26. Therefore, we asked whether other factors were involved in the regulation of the status H4R3me2s mediated by PRMT5. Given that WDR77 greatly enhanced the PRMT5's methyltransferase activity 25, we supposed that WDR77 might be required for the methylase activity of PRMT5 that repressed the cccDNA transcription. Strikingly, our data verified that WDR77 restricted cccDNA transcription and HBV replication in HBV *de novo* infection PHHs and HepG2-NTCP cells in a PRMT5 methyltransferase activity-dependent manner, leading to the increase of cccDNA-bound H4R3me2s. Our finding suggests that WDR77 is able to restrict the cccDNA transcription in the cells. PRMT5 has been reported to implicate in the regulation of viral replication and pathogenesis by targeting a variety of cellular substrates and viral proteins in a methyltransferase activity-dependent manner, such as HBV replication, bovine leukemia virus (BLV) infection, the human immunodeficiency virus type-1 (HIV-1) replication, the human T-cell lymphotropic virus type-1 (HTLV-1) replication, and the mouse mammary tumor virus (MMTV) replication and RNA/DNA virus infection^42^-^44^. However, whether WDR77 is involved in other viral regulation through modulating the activity of PRMT5 has not been reported.

We next examined the underlying mechanism by which HBx decreased the levels and stability of WDR77 protein. Given that HBx interacted with DDB1-CUL4-ROC1(CRL4) E3 ligase 34, we investigated whether HBx recruited WDR77 for ubiquitination and degradation by DDB1-CUL4-ROC1 E3 ligase. Interestingly, our data validated that HBx degraded WDR77 in a DDB1-HBx and proteasome-dependent manner. It suggests that HBx drives the cellular DDB1-containing E3 ubiquitin ligase to degrade WDR77 through recruiting WDR77, leading to the disability of methyltransferase activity of PRMT5. This conclusion agrees with the previously presented dataset by Decorsiere et al that WDR77 is only recovered in the HBx-DDB1 mutant fusion which cannot incorporate into the E3 ligase complex 19. Thus, we conclude that HBx attenuated the WDR77-mediated HBV repression by driving DDB1-induced WDR77 degradation in the liver. Therapeutically, WDR77 may be useful to identify the inhibitors of cccDNA.

**Figure 7:**
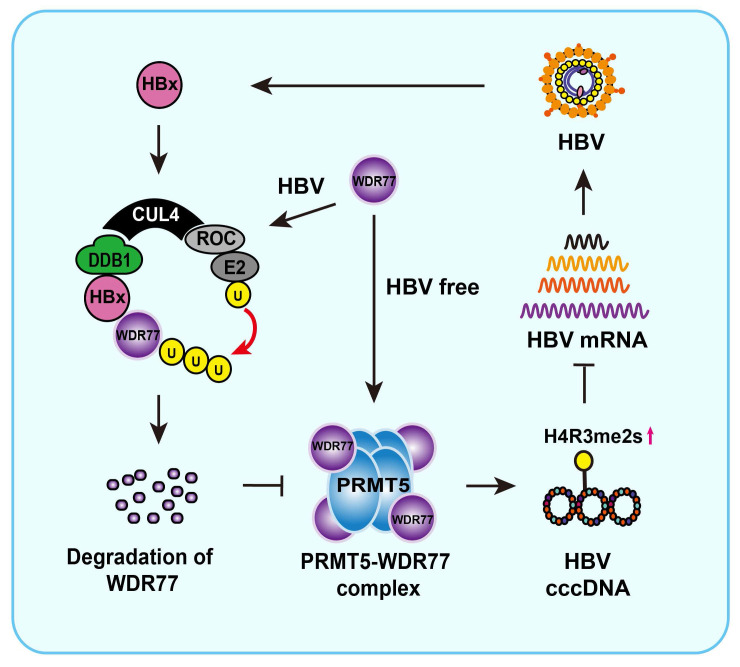
**A model of HBx degrading WDR77 to enhance HBV replication by DDB1-mediated WDR77 degradation in the liver.** In this model, HBx binds to DDB1 and recruits WDR77 for degradation by the DDB1-CUL4-ROC1 (CRL4) E3 ligase. WDR77 is able to enhance the methylase activity of PRMT5 which inhibits cccDNA transcription by increasing the cccDNA-bound H4R3me2s. The degradation of WDR77 mediated by HBx-DDB1 leads to the decrease of the cccDNA-bound H4R3me2s catalyzed by PRMT5, resulting in the activation of cccDNA transcription.

Overall, we summarize a model of HBx promoting cccDNA transcription by targeting WDR77 (Figure 7). In this model, HBx binds to DDB1 and recruits WDR77 for the degradation by DDB1-CUL4-ROC1 (CRL4) E3 ligase. WDR77 is able to enhance the methylase activity of PRMT5 which inhibits cccDNA transcription by increasing the levels of cccDNA-bound H4R3me2s. The degradation of WDR77 mediated by HBx-DDB1 leads to the decrease of the cccDNA-bound H4R3me2s catalyzed by PRMT5, resulting in the activation of cccDNA transcription. Our finding provides new insights into the mechanism by which HBx enhances HBV replication in the liver.

## Materials and Methods

### Cell lines and cell culture

HepG2 and HEK293T cells were purchased from the European Collection of Cell Cultures (ECCAC, Salisbury, UK). The dHepaRG cells were purchased from Biopredic International (Rennes, France). An expression plasmid for hNTCP was transfected into HepG2 cells with TransIT-LT1 (Mirus, USA) to establish HepG2-NTCP cells according to the manufacturer's instructions 7. HepG2-H1.3x-stable cell lines used to produce HBV virions carrying a defective HBx gene have been described 12, 45. HepG2 H1.3 (ΔX) cells were established by stable integration of a 1.3-fold HBV genome (genotype D, subtype ayw) carrying premature stop codon mutations in both the 5′ and 3′ HBx open reading frames. The HepG2 cell line stably expressing WDR77 (termed HepG2-WDR77) was generated by transfecting the plasmid of WDR77 using TransIT-LT1 (Mirus, USA) in HepG2 cells according to the manufacturer's instructions. HepG2, HepG2-NTCP, dHepaRG, HepG2-WDR77 and HEK293T cells were maintained in Dulbecco's modified Eagle's medium (Gibco, Grand Island, NY, USA) with 10% fetal bovine serum (Gibco, Grand Island, NY, USA). HepAD38 cell line was purchased from BioVector NTCC Inc. (Beijing, China). HBV replication could be regulated in HepAD38 cell line through the presence or absence of tetracycline in the culture medium 46. HepAD38 cell was cultured in DMEM/F12 medium (Life Technologies, Carlsbad, CA) supplemented with 10% FBS, 400 μg/mL G418, and with 0.3 μg/mL tetracycline (for inhibition of HBV replication) or without any tetracycline (for induction of HBV replication). PHHs were purchased from Shanghai RILD Inc. (Shanghai, China). The cells were cultured similarly using the same plating and incubation medium as described 7, 46. All the cell lines were treated with 100 U/mL penicillin, and 100 mg/mL streptomycin in a humidified incubator equilibrated with 5% CO_2_ at 37°C.

### *In vitro* methylation assays

The methyltransferase activity of PRMT5 was performed as described 47. Flag-PRMT5 was purified from transfected HepG2-WDR77 cells by anti-Flag tag immunoprecipitation. The immobilized PRMT5 proteins were then incubated with 20 μL of HMTase buffer (20 mM Tris, pH 8.8, 25 mM NaCl, 4 mM EDTA, 1 mM PMSF, 0.5 mM DTT) supplemented with 10 μg of purified histone H4 and 2 mCi 3H-SAM (Amersham, UK) at 30℃ for 2 h. The reaction was stopped by the addition of sample loading buffer for immunoblotting.

### HBV partials collection and infection

HBV partials used in this study were mainly derived from HepAD38 and HepG2 H1.3 (ΔX) cells. HBV particles were concentrated from the clarified supernatant by overnight precipitation with 10% PEG8000 and 2.3% NaCl. The precipitates were washed and resuspended with medium at 200-fold concentration. HBV DNA was quantified by real-time PCR to determine enveloped DNA-containing viral particles (vp). Only inocula reaching a titer between 10^9^ and 10^10^ vp/mL were used. HepG2-NTCP cells, dHepaRG, and PHHs were infected with HBV partials at a multiplicity of infection (MOI) of indicated amount of vp/cell in the presence of 4% PEG8000 at 37˚C for 16 h as previously described 46, 48.

### Patient samples

The liver tissues of the patients were intraoperatively harvested or obtained by needle biopsy that obtained from Tianjin Third Center Hospital (Tianjin, P.R. China). Clinicopathological information about the patients was obtained from patient records, and was summarized in Table S3. Written consents approving the use of their tissues for research purposes after operation were obtained from patients. The Institute Research Ethics Committee at the Nankai University approved the study protocol.

### Plasmid or siRNA generation and transfection

All the plasmids used in this study were constructed by Genscript Biotechnology Co., Ltd (Nanjing, China) unless specifically stated. The plasmids used in this study for construction were listed in Table S4. All siRNAs were synthesized by Sangon Biotech (Shanghai, China). The siRNA sequences used in this study were listed in Table S5. The cells were cultured in a 10-cm dish, 6-well plate, 24-well plate, or 96-well plate for 12 h and then were transfected with plasmid or siRNAs (Unless indicated, 100 nM for siRNA transfection, cells were transfected with 10, 2, 1, 0.2 μg plasmid cultured in 10-cm dish 6-well plate, 24-well plate, or 96-well plate). The transfections were performed by using Lipofectamine 3000 reagent (Invitrogen, Carlsbad, CA) according to the manufacturer's protocol.

### Generation of human liver-chimeric mice (Huhep-URG mice)

HBV-infected human liver-chimeric mice (HBV-Huhep-URG mice) containing the intact HBV genome and human liver-chimeric mice (Huhep-URG mice) without HBV infection were generated by VITALSTAR (Beijing, China). Briefly, PHHs were transplanted into 3-week-old urokinase-type plasminogen activator/severe combined immunodeficient beige mice (URG mice) by intrasplenic injection as described 49, 50. Engraftment and viability of PHHs were assessed by quantification of human serum albumin by Human Albumin ELISA kit (Immunology Consultants Lab, Portland, USA). Then, three Huhep-URG mice were infected with 2.5 × 10^8^ IU/mL (0.2 mL/mouse) HBV particles. Serum HBV DNA was determined in the mice by quantitative PCR (Da An Gene, Guangzhou, China). The information of human liver-chimeric mice was shown in Figure 1A and Table S1. The Huhep-URG mice and HBV-Huhep-URG mice were sacrificed 8 weeks after virus inoculation.

All animal experiments were in accordance with the Guide for the Care and Use of Laboratory Animals and were performed according to the institutional ethical guidelines. The Institute Research Ethics Committee at the Nankai University approved the study protocol.

### RNA extraction and quantitative real-time PCR (RT-qPCR)

Total RNA was extracted from cells or liver tissues from human liver-chimeric mice using Trizol reagent (Solarbio, Beijing, China). First-strand cDNA was synthesized using the Hifair Ⅲ 1st strand cDNA synthesis supermix kit (Yeasen Biotech, Shanghai, China), in which the 5×gDNA digester Mix could remove the residual genomic DNA contamination. Quantitative real-time PCR was performed on StepOnePlus real-time PCR machine (Bio-Rad), using qPCR SYBR Green Master Mix (Yeasen Biotech, Shanghai, China). RT reaction without the enzyme followed by qPCR was used as a RT control in the experiments. Relative transcriptional folds were calculated as 2^-ΔΔCt^. The primers used were listed in Table S6.

### Western blot analysis

Total protein lysates were extracted from hepatoma cells or liver tissues with RIPA buffer according to the manufacturer's protocol. Histones were extracted using the Histone Extraction Kit (Abcam, Cambridge, UK). Protein concentrations were measured using the Bradford Assay, and 20-50 μg protein extracts were subjected to SDS-PAGE. Then proteins were transferred to a nitrocellulose membrane, blocked with 5% non-fat milk and incubated with first antibodies for 1 h at 37℃. After incubation with secondary antibody against mouse (1:10,000) or rabbit (1: 10,000) for 1 h at 37℃, the membrane was visualized by Super ECL Detection Reagent (Yeasen Biotech, Shanghai, China). The antibodies used for Western blot analysis were listed in Table S7.

### Co-immunoprecipitation (CoIP) assays

Cells were washed with cold phosphate-buffered saline (PBS) and lysed with cold cell lysis buffer for 30 min at 4°C. Then, 500 μg of cellular extract was incubated with appropriate specific antibodies or normal rabbit/mouse immunoglobin G (IgG) at 4°C overnight with constant rotation, followed by the addition of protein A/G Sepharose beads and incubation for 2 h at 4°C. Beads were then washed five times with wash buffer (20 mM Tris-HCl, pH 7.5, 150 mM NaCl, 20 mM KCl, 1.5 mM MgCl_2_, 15% glycerol, 1 mM EDTA, 0.5% NP-40, 1% protease inhibitor). The immune complexes were subjected to SDS-PAGE followed by immunoblotting with the indicated antibodies. The antibodies used were listed in Table S7.

### HSA, HBsAg, HBeAg and HBV DNA quantification

Human serum albumin (HSA), hepatitis B virus surface antigen (HBsAg) and hepatitis B virus e antigen (HBeAg) in culture supernatants were assayed by commercial ELISA (Cayman, MI, USA; Kehua bio-engineering, Shanghai, China). HBV DNA was quantified in the supernatants of HepAD38 cells, HBV *de novo* infection HepG2-NTCP cells, HBV *de novo* infection PHH and HepG2 cells transfected with pCH-9/3091 using a diagnostic kit (Sansure Biotech, Hunan, China).

### HBV cccDNA isolation and Southern blot analysis

For nuclei isolation, cells were lysed in 500 μL of homogenization buffer (1 mM EDTA, 0.2% NP-40, 150 mM NaCl, 50 mM Tris-HCl, pH 8.0), and centrifuged for 10 min at 10,000 rpm at 4°C (Sorvall HB-4 rotor). Nuclei were then treated with 500 μL of lysis buffer (6% SDS, 100 mM NaOH), and the reaction was mixed completely and incubated for 30 min at 37°C. After neutralization with 3 mol of sodium acetate (pH 5.2) lysates were cleared for 20 min at 10,000 rpm at 4°C. HBV DNA was extracted from the supernatant with phenol/chloroform, precipitated with ethanol, and dissolved in 50 μL of 10 mM Tris-HCl (pH 7.5) and 1 mmol EDTA (pH 8.0), and digested with plasmid-safe ATP-dependent DNase (Epicentre). The qPCR was performed using HBV cccDNA specific primers as described 49. The cccDNA specific primers span the gap and the nick in the rcDNA form of the HBV genome and were designed not to detect rcDNA or a linear HBV genome. Using optimized PCR conditions, the primers was determined the specificity to amplify cccDNA over rcDNA to be 10^3^ to 1 49. For Southern blot analysis, the extracted HBV cccDNA sample was separated through 1.0% agarose gel, blotted onto a nylon membrane, and hybridized with DIG-labeled probes of linear HBx DNA fragments.

### Immunofluorescence assays

Cells in 6-well plates were washed three times with pre-cooled PBS and fixed by 4% paraformaldehyde for 10 min, followed by permeabilization for 10 min at room temperature with 0.5% Triton X-100. After incubation for 1 h with 5% BSA for blockade of nonspecific binding, primary antibodies were added for incubation overnight at 4°C. The bound antibodies were visualized by incubation with secondary antibodies. Images were acquired using a confocal microscope or a fluorescence microscopy. The antibodies used were listed in Table S7.

### Chromatin immunoprecipitation (ChIP) and Re-ChIPs

ChIP experiments were performed by using simple CHIP(R) plus sonication CHIP kit 4C and RT Reagents (Cell signaling, MA, USA) 38, 51. After the reverse crosslinking, DNA was extracted and further treated with Plasmid-Safe ATP-Dependent DNase (Epicentre Biotechnologies, Madison, WI) to degrade contaminating RC and single-stranded forms of HBV DNA 51. Re-ChIP assays were performed by using Re-ChIP-IP^R^ kit (Active Motif, Shanghai, China). Briefly, the immunoprecipitated chromatin was removed from the magnetic beads. The chromatin was then desalted and a second ChIP was performed using a different antibody from the first. The cross-licks of these sequentially immunoprecipitated protein-DNA complexes were then reversed and the DNA was analyzed by qPCR. The primers used are listed in Table S6. Samples were normalized to input DNA using the ΔCt method and calculated as percentage of the input, Enrichment percentage = 2% × 2 ^Ct (input) -Ct (IP sample)^.

### Immunohistochemistry (IHC) staining

The HBV-infected liver tissue and uninfected liver tissue slices were obtained from the VITALSTAR (Beijing, China). First, the samples were dewaxed. Then, the antigen retrieval was applied at 95 °C with citrate buffer (pH 6.0) for 15 min. The slides were treated with 3% H_2_O_2_ for 10 min and blocked with goat serum for 1 h. Then, slides were incubated with the monoclonal antibody at 4 °C overnight. After washing three times with 0.01 M PBS, samples were incubated with goat anti‐rabbit or anti‐mouse IgG coupled to horseradish peroxidase (ORIGENE, Beijing, China) for 30 min at 37 °C. Immunostaining was proceeded using chromogen 3, 3′-Diaminobenzidine (DAB), and counter stained with Mayer's hematoxylin (ZSBG-BIO, China). The slices were then dehydrated and covered with a coverslip. The antibodies used for IHC staining were listed in Table S7.

### Pull-down assays

Recombinant His-HBx proteins were purified from E.coli with Ni-NTA resin (GE Healthcare, Waukesha, WI) performed as described 52. In brief, His-tagged HBx purified from inclusion bodies consists of four major steps: isolation of purified inclusion bodies, solubilization of inclusion bodies, refolding of solubilized proteins and purification of refolded proteins by various chromatographic techniques. HEK293T cells were transfected with Flag-tagged WDR77 plasmids using Lipofectamine 3000 reagent (Invitrogen, Carlsbad, CA, USA) according to the manufacturer's protocol. Cells were harvested after transfection and homogenized in lysis buffer. After centrifugation at 21,000 g for 10 min, the supernatants of cell lysates were incubated with anti-DDDDK-tag mAb-magnetic agarose (Medical & Biological Laboratories, Japan) for 2 h or overnight with gentle agitation. Then, the agarose beads and their binding proteins were washed four times with lysis buffer. Moreover, the agarose beads with Flag-WDR77 were incubated with 10 μg purified His-HBx at 4 °C overnight. The supernatants were collected as input and the beads were extensively washed 6 times with lysis buffer and resuspended in SDS loading buffer and boiled. The sample buffer was loaded in 12% SDS-PAGE for detection with the anti-His antibody. Standard immunoblotting procedures were followed using anti-Flag antibody and anti-His antibody.

### Northern blot analysis

Northern blot analysis was performed on total RNA isolated using TRIzol Reagent (Gibco-Invitrogen, UK) and treated with RNase-Free DNase I (Ambion, Life Technologies, USA). The RNA (10 μg per sample) was denatured by glyoxal treatment and separated on a 1% agarose gel using a Northern blot kit (Roche, USA). After capillary transfer to Hybond N+ membranes (Amersham, UK), the RNA was fixed on the membrane by ultraviolet cross-linking and hybridized with a DIG-labelled HBV genomic DNA probes.

### Statistical analysis

Each experiment was repeated at least three times unless otherwise indicated. All analyses and graphs were generated with GraphPad Prism 6. Statistical significance was assessed by comparing mean values (± SD) using a Student's *t* test for independent groups and was assumed for *p < 0.05; **p < 0.01; ***p < 0.001.

## Supplementary Material

Supplementary figures and tables.

## Abbreviations

CBP: cAMP response element binding protein (CREB) binding protein
cccDNA: covalently closed circular DNA
aDMA: asymmetric dimethylarginine
CHB: chronic hepatitis B
CHX: Cycloheximide
ChIP: chromatin immunoprecipitation
CoIP: coimmunoprecipitation
DDB1: Damage-specific DNA-binding protein 1
ELISA: enzyme-linked immuno sorbent assay
GCN5: general control nonderepressible 5
H4R3me2s: symmetric dimethylation of arginine 3 on H4
HAT1: histone acetyltransferase 1
HBc: HBV core protein
HBx: hepatitis B x protein
HCC: hepatocellular carcinoma
HDAC1: histone deacetylase 1
IHC: Immunohistochemistry
MMA: monomethyl arginine
PCAF: p300/CREB binding protein associated factor
RC-DNA: relaxed circular DNA
pgRNA: pregenome RNA
Rme2sy: symmetrical methylation of arginine residues
PHHs: primary human hepatocytes
PRMT5: protein arginine methyltransferases 5
PRMTs: protein arginine methyltransferases
PPIs: protein-protein interactions
PTMs: post-translational modifications
RT-qPCR: reverse transcription quantitative real-time polymerase chain reaction
sDMA: symmetric dimethylarginine
SD: standard deviation
SMC: structural maintenance of chromosomes
siRNA: small interfering RNA
SIRT1: silent information regulator 2 homolog 1
WDR77: WD repeat domain 77 protein
