# Supplementary material for: HBx represses WDR77 to enhance HBV replication by DDB1-mediated WDR77 degradation in the liver
Source: Theranostics. 2021 Jul 25;11(17):8362–78. doi: 10.7150/thno.57531 (PMC8343998; doi:10.7150/thno.57531)
Supplement: Supplementary file 1 — Supplementary figures and tables. [file thnov11p8362s1.pdf]

Supplementary Figures

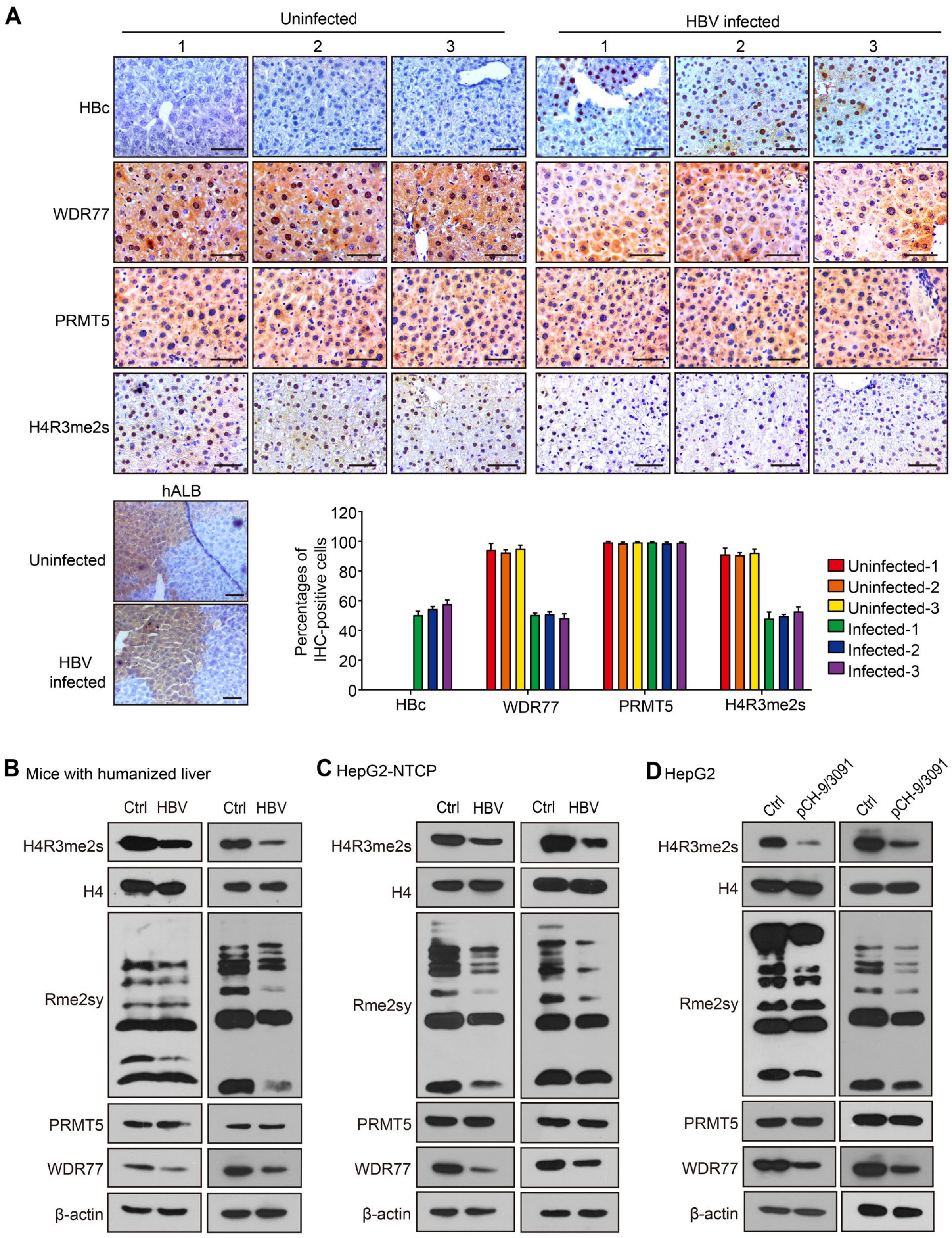

Figure S1. HBV infection leads to the decrease of PRMT5 methylase activity and WDR77 level.

(A) Immunohistochemistry assays for HBc, WDR77, PRMT5, H4R3me2s, and human-albumin (hALB) were performed in the liver tissues of human liver-chimeric mice. Representative pictures of IHC are shown. The graph with quantification information of the staining of all the mice is shown (down, right).

(B) The levels of H4R3me2s, Rme2sy, PRMT5, and WDR77 were examined by Western blot analysis in the liver tissues of human liver-chimeric mice, respectively. The data are representative of two repeats.

(C) HepG2-NTCP cells were uninfected or infected with wild-type HBV (at a multiplicity of infection of 1000 vp/cell). The levels of H4R3me2s, Rme2sy, PRMT5, and WDR77 were tested by Western blot analysis in the cells 7 days later, respectively. The data are representative of two repeats.

(D) HepG2 cells were transfected with pCH9 (Vector control) or pCH9/3091 (HBV-expressing) plasmids. The levels of H4R3me2s, Rme2sy, PRMT5, and WDR77 were evaluated by Western blot analysis 3 days later, respectively. The data are representative of two repeats.

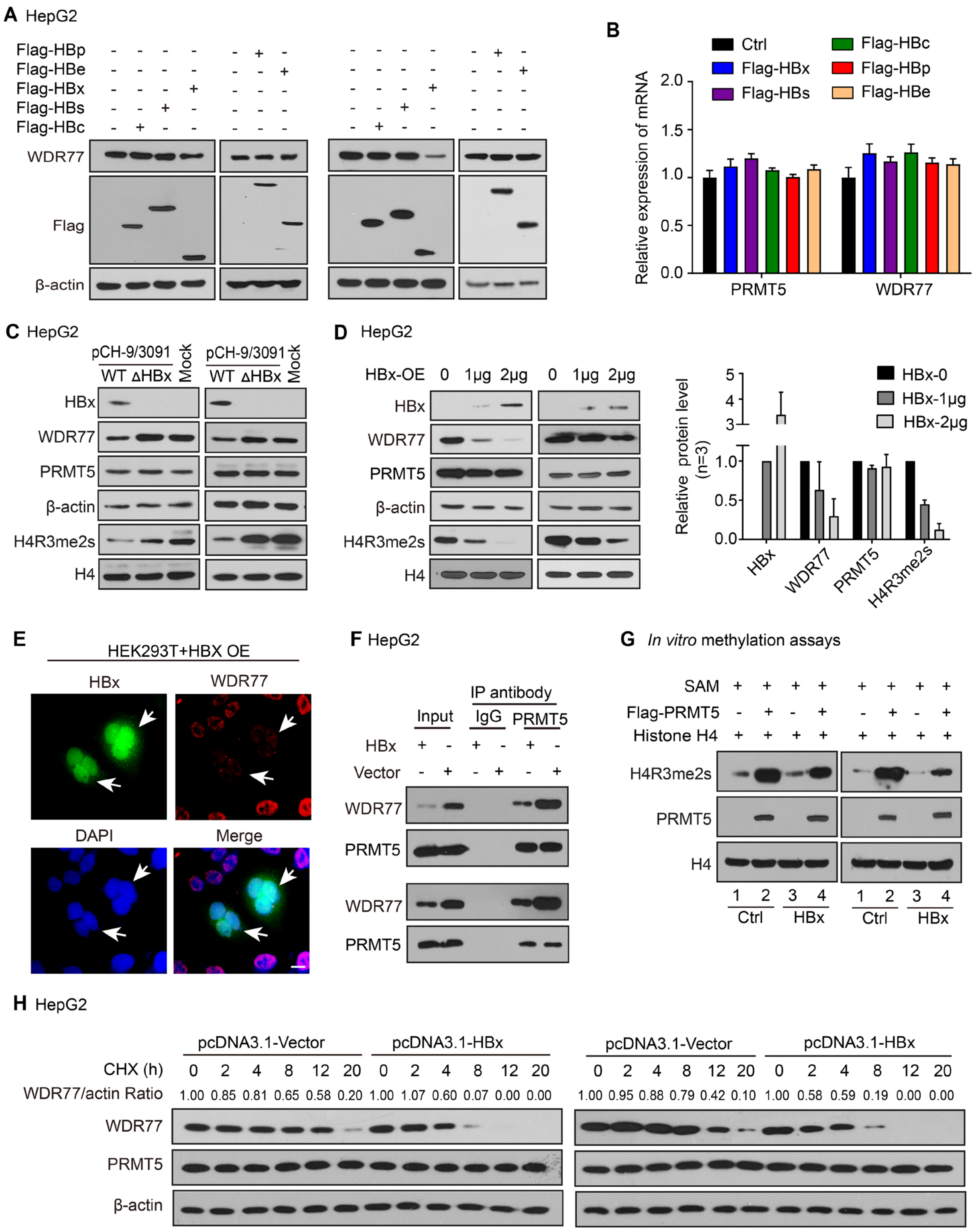

Figure S2. HBx decreases the PRMT5 methylase activity and WDR77 level.

(A) HepG2 cells were transfected with Flag-tagged HBp, HBe, HBx, HBs, and HBc plasmids, respectively. The effect of HBp, HBe, HBx, HBs, and HBc on WDR77 was detected by Western

blot analysis in the cells 3 days later. The data are representative of two repeats.

(B) HepG2 cells were transfected with Flag-tagged HBx, HBs, HBc, HBp, and HBe plasmids, respectively. The mRNA levels of WDR77 and PRMT5 were detected by RT-qPCR in the cells 3 days later.

(C) HepG2 cells were transfected with pCH-9/3091 (WT) or HBx-deficient pCH-9/3091(△HBx) plasmids. The levels of HBx, WDR77, PRMT5 and H4R3me2s were tested by Western blot analysis in the cells 3 days later. The data are representative of two repeats.

(D) HepG2 cells were transfected with pcDNA3.1-HBx (2 μg) or pcDNA3.1-Vector plasmids (2 μg). The levels of HBx, WDR77, PRMT5, and H4R3me2s were measured by Western blot analysis in the cells 3 days later, respectively. The data are representative of two repeats. The quantification of the Western blot analysis for 3 experiments (the other test in Figure 2D) was shown (down).

(E) HEK293T cells were transfected with Flag-tagged HBx plasmids. The expression of WDR77 (red) and HBx (green) was assessed by immunofluorescence assays in the cells 3 days later.

(F) HepG2 cells were transfected with pcDNA3.1-HBx or pcDNA3.1-Vector plasmids. PRMT5 was immunoprecipitated by anti-PRMT5 antibody in the cells, and the levels of WDR77 was analyzed by CoIP analysis 3 days later. The data are representative of two repeats.

(G) HepG2-WDR77 cells were co-transfected with Flag-PRMT5 and pcDNA3.1-HBx or pcDNA3.1-Vector plasmids. An *in vitro* methylation assays were performed by using Flag-PRMT5 purified from the cells 3 days later. The data are representative of two repeats.

(H) HepG2 cells were transfected with pcDNA3.1-HBx or pcDNA3.1-Vector 48 h, followed by the treatment with cycloheximide (CHX) (100 μg/mL) according to the indicated time. The

protein levels of WDR77 and PRMT5 were detected by Western blot analysis in the cells. The data are representative of two repeats.

Data are represented as means ± SD (n = 3). Student's *t* test, *$p < 0.05$; **$p < 0.01$; ***$p < 0.001$.

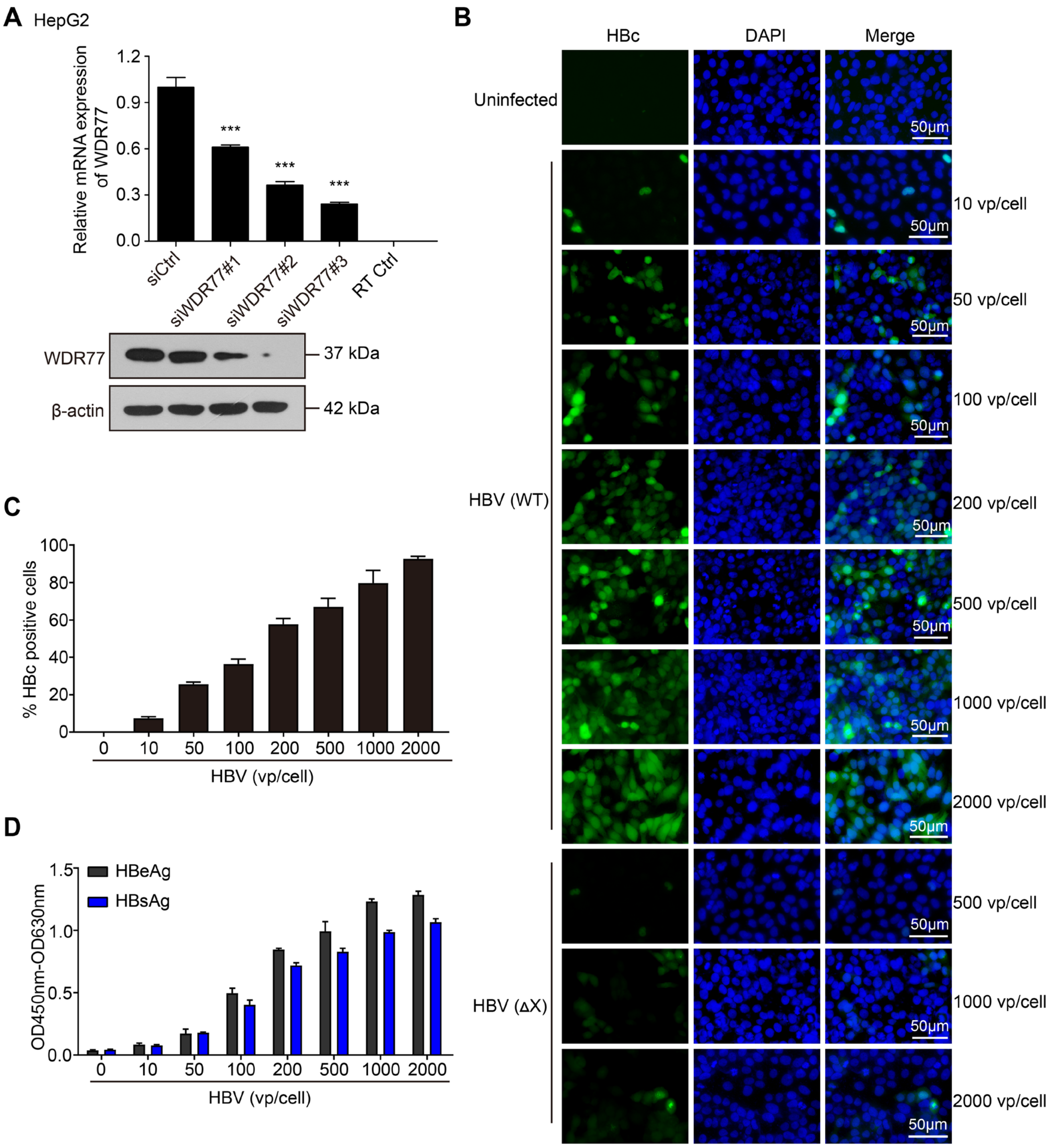

Figure S3. WDR77 represses the HBV replication.

(A) HepG2 cells were transfected with siRNA of WDR77. The knockdown efficiency of siWDR77 was examined by RT-qPCR and Western blot analysis 3 days later, respectively. RT Ctrl means the reaction without the enzyme followed by qPCR.

(B) The expression of HBc was examined by immunofluorescence analysis in PHH cells infected with wild-type (WT) or HBx-deficient (ΔX) HBV at the indicated viral genome equivalents per

cell 7 days after infection.

(C) Quantification of the immunofluorescence images. Data are mean ± SD of at least three fields.

(D) PHH cells were infected (or uninfected) with wild-type HBV particles at the indicated viral-genome equivalents per cell. The levels of HBeAg and HBsAg in the supernatants of cells were assessed by ELISA 7 days later.

Data are represented as means ± SD (n = 3). Student's *t* test, ns, no significant; *$p < 0.05$; **$p < 0.01$; ***$p < 0.001$.

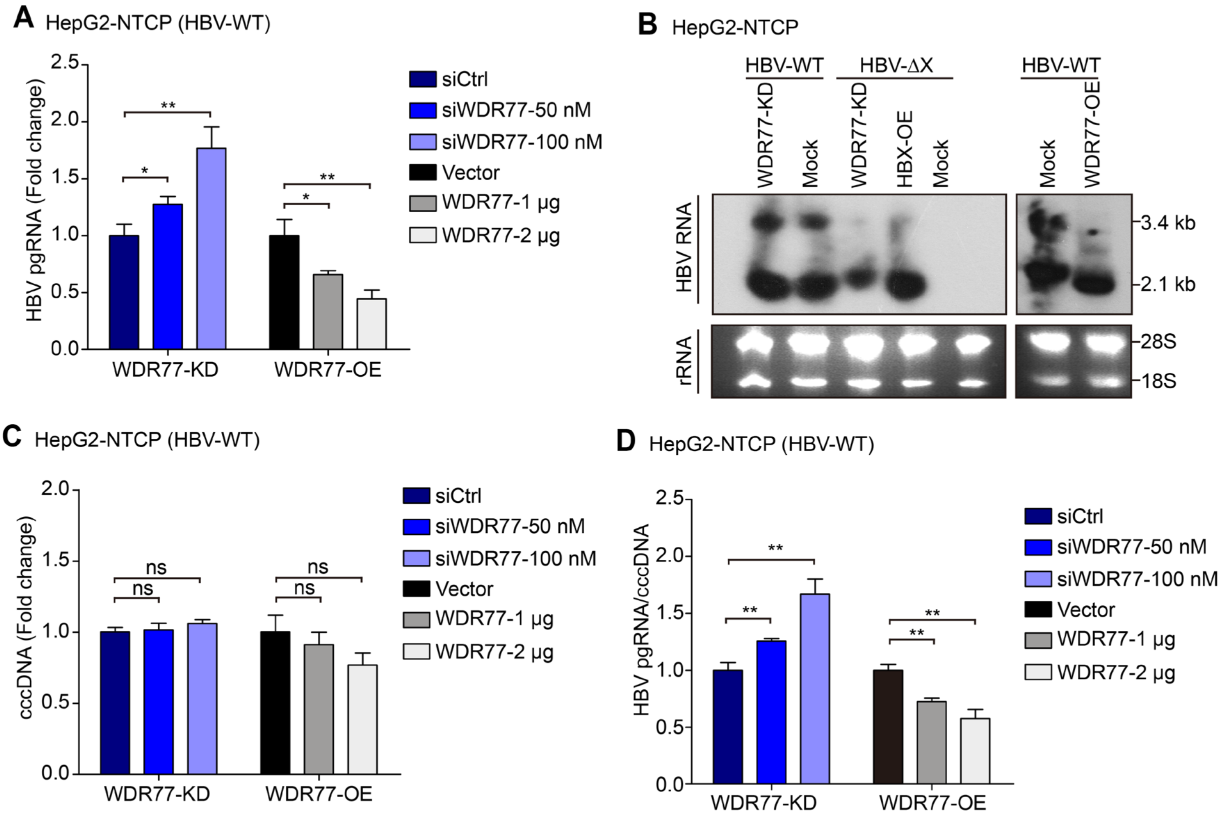

Figure S4. WDR77 attenuates the transcription activity of cccDNA.

(A) HepG2-NTCP cells were infected with wild-type HBV (at a multiplicity of infection of 1000 vp/cell) and were continuously transfected with plasmids or siRNA of WDR77 at -2, 1, and 4 dpi (days post-infection). The levels of HBV pgRNA were quantified by RT-qPCR assays in the cells 7 days post-infection.

(B) HepG2-NTCP cells were infected with wild-type or HBx-deficient HBV (at a multiplicity of infection of 1000 vp/cell) and were continuously transfected with pcDNA3.1-HBx, siWDR77 or pcDNA3.1-WDR77 at -2, 1, and 4 dpi (days post-infection). The levels of HBV RNA were examined by Northern blot analysis in the cells 7 days post-infection.

(C) HepG2-NTCP cells were infected with wild-type HBV (at a multiplicity of infection of 1000 vp/cell) and were continuously transfected with siWDR77 or pcDNA3.1-WDR77 at -2, 1, and 4 dpi (days post-infection). The levels of cccDNA were tested by RT-qPCR analysis in the cells 7

days post-infection.

(D) HBV transcription activity was assessed by calculating the ratio of pgRNA/cccDNA.

Data are represented as means ± SD (n = 3). Student's *t* test, ns, no significant; *$p < 0.05$; **$p < 0.01$; ***$p < 0.001$.

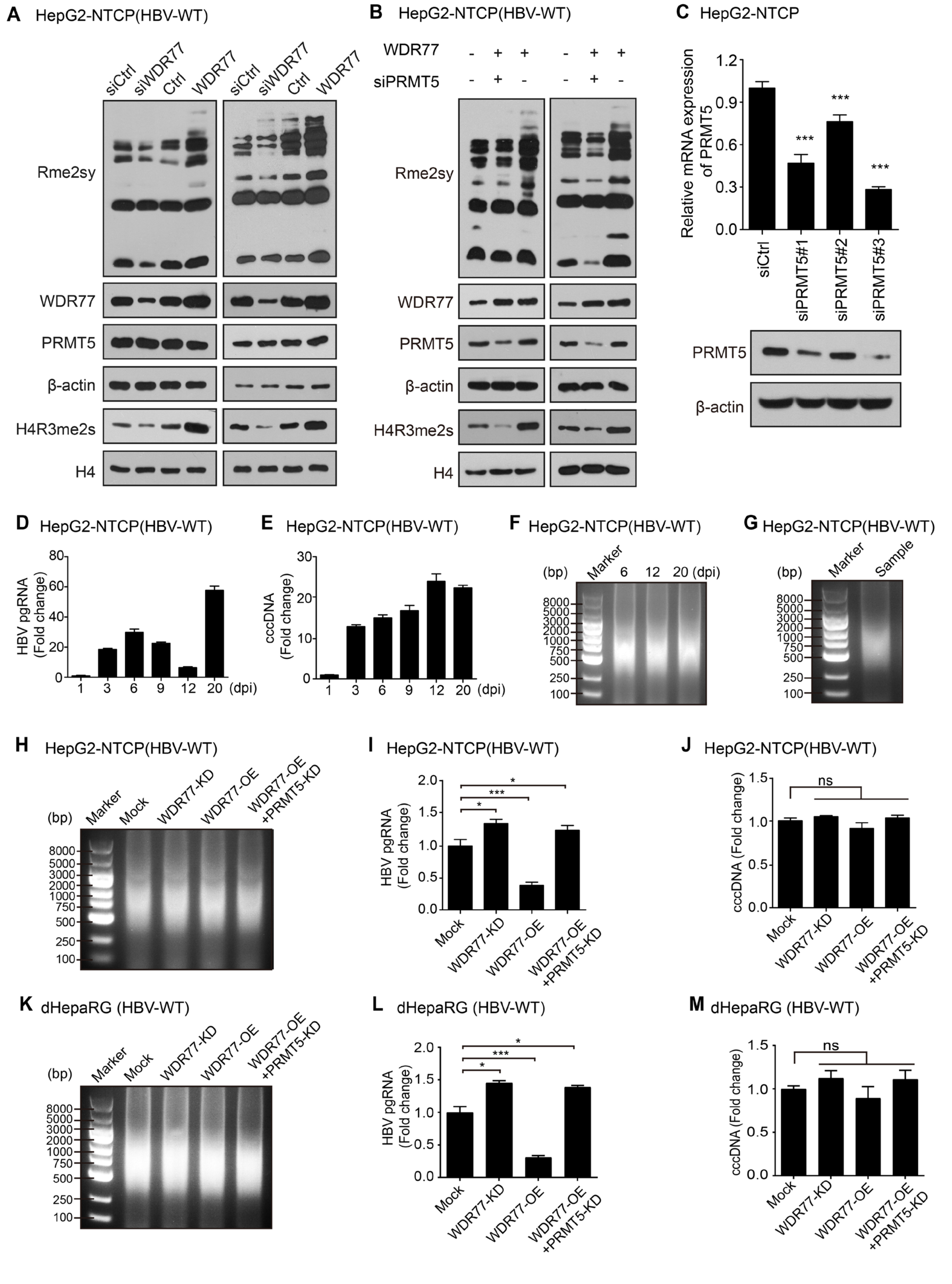

Figure S5. WDR77 is required for the PRMT5-mediated inhibition of cccDNA transcription.

(A) HepG2-NTCP cells were infected with wild-type HBV (at a multiplicity of infection of 1000

vp/cell) and were continuously transfected with siWDR77 or pcDNA3.1-WDR77 at -2, 1, and 4 dpi (days post-infection). The levels of Rme2sy, WDR77, PRMT5 and H4R3me2s were detected by Western blot analysis in the cells 7 days post-infection, respectively. The data are representative of two repeats.

(B) HepG2-NTCP cells were infected with wild-type HBV (at a multiplicity of infection of 1000 vp/cell) and were continuously transfected with siPRMT5 or pcDNA3.1-WDR77 at -2, 1, and 4 dpi (days post-infection). The levels of Rme2sy, WDR77, PRMT5 and H4R3me2s were evaluated by Western blot analysis in the cells 7 days post-infection, respectively. The data are representative of two repeats.

(C) HepG2-NTCP cells were transfected with siRPMT5. The knockdown efficiency of siPRMT5 was examined by RT-qPCR and Western blot analysis in the cells 3 days later.

(D) HepG2-NTCP cells were infected with wide-type HBV (at a multiplicity of infection of 1000 vp/cell). The levels of HBV pgRNA were quantified by RT-qPCR assays in the cells at indicated days. The dpi means days post-infection.

(E) HepG2-NTCP cells were infected with wide-type HBV (at a multiplicity of infection of 1000 vp/cell). The levels of cccDNA were examined by qPCR in the cells at indicated days. The dpi means days post-infection.

(F) The figures of the chromatin fragments after sonication in Chip assays (Fig. 5D) were showed.

(G) The figures of the chromatin fragments after sonication in Chip assays (Fig. 5E-F) were showed.

(H) The figures of the chromatin fragments after sonication in Chip assays (Fig. 5G) were showed.

(I) The levels of HBV pgRNA were tested by RT-qPCR assays in HBV *de novo* infection HepG2-

NTCP cells transfected with siWDR77, pcDNA3.1-WDR77 or co-transfected with pcDNA3.1-WDR77 and siPRMT5.

(J) The levels of cccDNA were evaluated by qPCR in HBV *de novo* infection HepG2-NTCP cells transfected with siWDR77, pcDNA3.1-WDR77 or co-transfected with pcDNA3.1-WDR77 and siPRMT5.

(K) The figures of the chromatin fragments after sonication in Chip assays (Fig. 5I) were showed.

(L) The levels of HBV pgRNA were tested by RT-qPCR assays in HBV *de novo* infection dHepaRG cells transfected with siWDR77, pcDNA3.1-WDR77 or co-transfected with pcDNA3.1-WDR77/siPRMT5, respectively.

(M) The levels of cccDNA were evaluated by qPCR in HBV *de novo* infection dHepaRG cells transfected with siWDR77, pcDNA3.1-WDR77 or co-transfected with pcDNA3.1-WDR77 and siPRMT5.

Data are represented as means ± SD (n = 3). Student's *t* test, ns, no significant; *$p < 0.05$; **$p < 0.01$; ***$p < 0.001$.

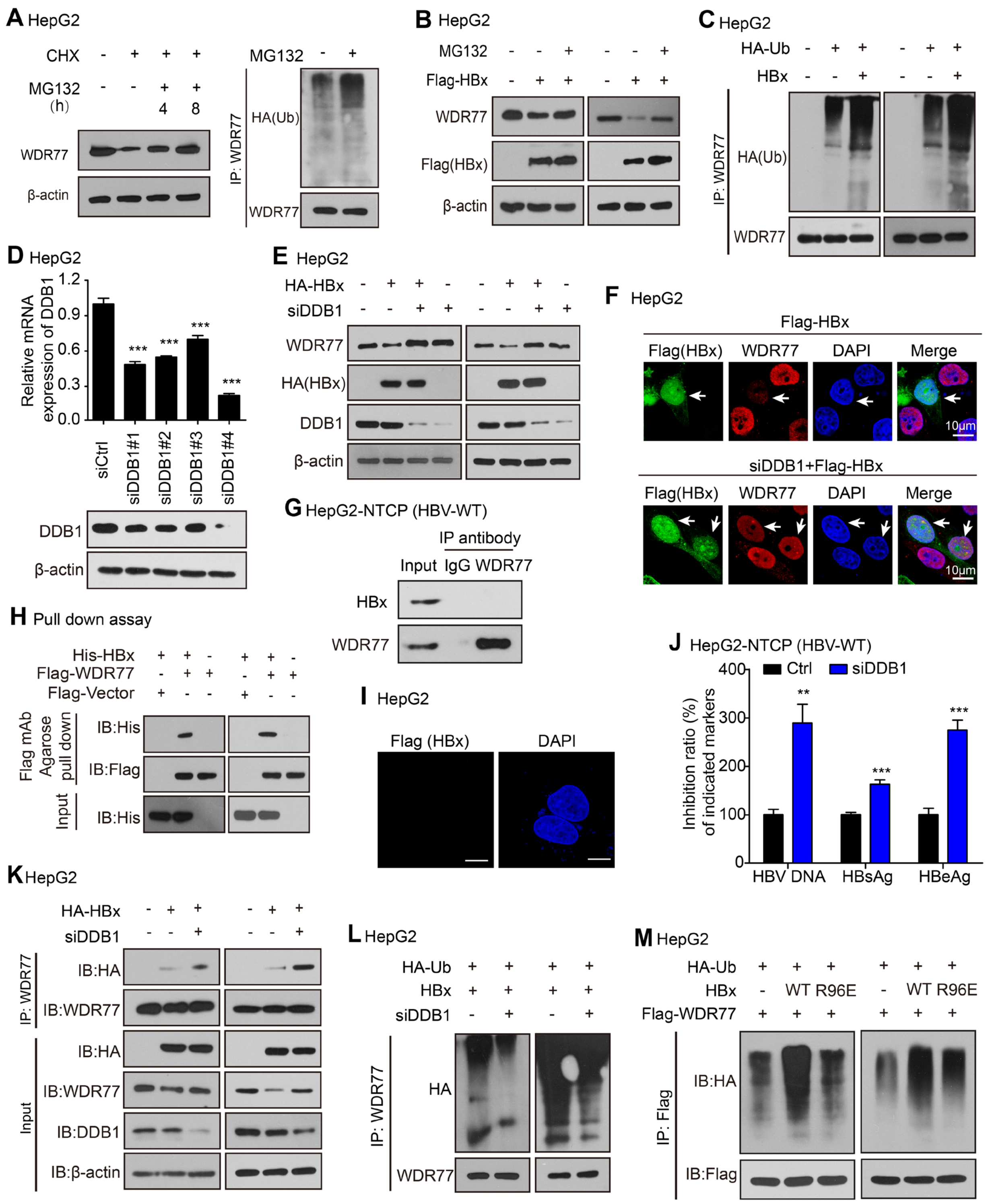

Figure S6. HBx degrades WDR77 by DDB1-CUL4-ROC1 E3 ligase.

(A) The protein levels of WDR77 were detected by Western blot analysis in HepG2 cells treated with MG132 (2 mM, 4 h or 8 h) and/or cycloheximide (CHX) (100 μg/mL, 20 h) (Left). The ubiquitylation (Ub) of immunoprecipitated WDR77 was examined by Western blot analysis in

HepG2 cells treated with MG132 (2 mM, 8 h) (Right).

(B) HepG2 cells were transfected with Flag-tagged HBx for 48 h and then treated with MG132 (2 mM) for 24 h. The protein levels of WDR77 were detected by Western blot analysis in the cells. The data are representative of two repeats.

(C) HepG2 cells were transfected with the indicated plasmids (pcDNA3.1-HBx and HA-tagged Ubiquitin) and treated with MG132 (2 mM) 4 h before harvest. The ubiquitylation (Ub) of immunoprecipitated WDR77 was examined by Western blot analysis in the cells 3 days later. The data are representative of two repeats.

(D) HepG2 cells were transfected with siDDB1. The knockdown efficiency of siDDB1 was examined by RT-qPCR and Western blot analysis in the cells 3 days later.

(E) HepG2 cells were transfected with HA-tagged HBx and/or siDDB1. The protein levels of WDR77, HA (HBx) and DDB1 were measured by Western blot analysis in the cells 3 days later. The data are representative of two repeats.

(F) HepG2 cells were co-transfected with Flag-tagged HBx and siCtrl or siDDB1. The Flag-tagged HBx (green) and WDR77 (red) was determined by immunofluorescence staining in the cells 3 days later. Scale bars, 10 μm.

(G) HepG2-NTCP cells were infected with wild-type HBV (at a multiplicity of infection of 1000 vp/cell). CoIP assays were used to test the binding of HBx to WDR77 in the cells.

(H) His-tagged HBx was purified from E.coli with Ni-NTA resin. Flag-tagged WDR77 was purified from HEK293 cells transfected with Flag-tagged WDR77 plasmid with anti-DDDDK-tag mAb-magnetic agarose. The agarose beads with Flag-WDR77 were incubated with 10 μg purified His-HBx at 4 °C overnight and immunoblotting with anti-His antibody. The data are

representative of two repeats.

(I) The Flag-tagged HBx (green) was determined using anti-Flag antibody by immunofluorescence staining in HepG2 cells. Scale bars, 10 μm.

(J) The levels of HBV DNA (HBsAg and HBeAg) were tested by real-time PCR (ELISA) in HBV infected HepG2-NTCP cells transfected with siControl or siDDB1.

(K) HepG2 cells were transfected with HA-tagged HBx and/or siDDB1. The binding of HBx to immunoprecipitated WDR77 was examined by Western blot analysis in the cells 3 days later. The data are representative of two repeats.

(L) HepG2 cells were co-transfected with pcDNA3.1-HBx, HA-tagged Ubiquitin and siDDB1. And then treated with MG132 (2 mM) 4 h before harvest. Ubiquitylation of immunoprecipitated WDR77 was tested by Western blot analysis in the cells 3 days later. The data are representative of two repeats.

(M) HepG2 cells were co-transfected with pcDNA3.1-HBx/pcDNA3.1-HBx mut, HA-tagged Ubiquitin and Flag-tagged WDR77. And then treated with MG132 (2 mM) 4 h before harvest. Ubiquitylation of immunoprecipitated WDR77 was evaluated by Western blot analysis in the cells 3 days later. The data are representative of two repeats.

Data are represented as means ± SD (n = 3). Student's t test, *$p < 0.05$; **$p < 0.01$; ***$p < 0.001$.

Supplementary Tables

Table S1. The information of mice with humanized liver.

**Mice**	**Humanization (%)**	**HSA (μg /mL)**	**HBV DNA (×10^6 copy/mL)**
Mice-Uninfected-1	About 50	1916	0
Mice-Uninfected-2	About 50	2065	0
Mice-Uninfected-3	About 60	3243	0
Mice-HBV infected-1	About 40	1094	4.38
Mice-HBV infected-2	About 50	1571	23.7
Mice-HBV infected-3	About 40	631	32.6

Abbreviations: "HSA" refers to "Human Serum Albumin".

Table S2. The detection of HBsAg and HBV DNA in the supernatants of cells.

Samples	HBsAg (OD450-630) Mean ± SD	HBV DNA ($\times 10^7$ copy/mL) Mean ± SD
HepG2-NTCP uninfected	0.051 ± 0.004	0.00 ±0.00
HepG2-NTCP HBV-infected	0.953 ± 0.017	2.81 ± 0.26
HepG2-Ctrl	0.052 ± 0.002	0.00 ±0.00
HepG2-pCH-9/3091	0.693 ± 0.018	1.89 ± 0.25

Table S3. The characteristics of patients with biopsy.

Case No.	Age (yr)	Gender	Diagnosis	Organ	HBV (copy/mL)
1	58	Male	Viral hepatitis type B	Liver	8.29×10^{7}
2	58	Male	Viral hepatitis type B	Liver	1.57×10^{8}
3	68	Male	Viral hepatitis type B	Liver	1.93×10^{7}
4	36	Male	Viral hepatitis type B	Liver	1.08×10^{7}
5	73	Female	Viral hepatitis type B	Liver	7.50×10^{5}
6	57	Male	Viral hepatitis type B	Liver	2.60×10^{5}
7	68	Male	NA	Liver	$< 5.6\times10^{2}$
8	70	Male	NA	Liver	0
9	68	Male	NA	Liver	$< 5.6\times10^{2}$
10	74	Male	NA	Liver	$< 5.6\times10^{2}$
11	68	Male	NA	Liver	0
12	65	Male	NA	Liver	$< 5.6\times10^{2}$
13	64	Male	Viral hepatitis type B	Liver	1.72×10^{5}
14	59	Male	Viral hepatitis type B	Liver	6.89×10^{5}
15	71	Male	Viral hepatitis type B	Liver	4.52×10^{5}
16	65	Male	Viral hepatitis type B	Liver	1.16×10^{5}
17	55	Male	NA	Liver	$< 5.6\times10^{2}$
18	67	Male	NA	Liver	$< 5.6\times10^{2}$
19	59	Male	NA	Liver	$< 5.6\times10^{2}$
20	69	Male	NA	Liver	$<5.6\times10^{2}$

Abbreviations: "yr" refers to year. "NA" means not viral hepatitis type B.

Table S4. List of plasmids used in this paper.

Plasmids	Note
pcDNA3.1-HA-PRMT5	HA-tagged PRMT5
PCMV-3Taq-1a-PRMT5	Flag-tagged PRMT5
pcDNA3.1-HA-Vector	Vector
PCMV-3Taq-1a-Vector	Vector
pcDNA3.1-HA-WDR77	HA-tagged WDR77
PCMV-3Taq-1a-WDR77	Flag-tagged WDR77
pcDNA3.1-HA-HBx	HA-tagged HBx
PCMV-3Taq-1a-HBx	Flag-tagged HBx
pcDNA3.1a-PRMT5	PRMT5 (no tag)
pcDNA3.1a-WDR77	WDR77 (no tag)
pcDNA3.1a-Vector	Vector
pcDNA3.1a-HBx	HBx (no tag)
pcDNA3.1a-HBx (R96E)	HBx-mutant (R96E)
Pch-9/3091	1.1 copy wild type HBV plasmid
pCH-9/3091 mutant	HBx-deficient HBV plasmid
pcDNA3.1-HA-Ub	HA-tagged Ub
PCMV-3Taq-1a-HBs	Flag-tagged HBs
PCMV-3Taq-1a-HBc	Flag-tagged HBc

Table S5. List of siRNAs used in this paper.

Gene	Target sequence (Sense)
WDR77#1	GUGGACACCAAGAGUACAA
WDR77#2	CAAGCCUUUCUGAGUUGUUUA
WDR77#3	GGGAACUAGAUGAGAAUGA
Control	UUCUCCGAACGUGUCACGU
DDB1#1	GAGAAGAGGUGGAGGUGCA
DDB1#2	UGAUAAUGGUGUUGUGUUU
DDB1 #3	CCUGUUGAUUGCCAAAAAC
DDB1 #4	UAACAUGAGAACUCUUGUC
PRMT5#1	CCAGAAGAGGAGAAGGAUA
PRMT5#2	GGAUAAAGCUGUAUGCUGU
PRMT5#3	CCUCCAAGCUGUACAAUGA

Table S6. List of primers used in this paper.

Gene	Primer	Sequence (5'-3')
PRMT5	Forward	CTCTCAGTACCAGCAGGCCATC
	Reverse	GCGTCACCACGGCATTTG
GAPDH	Forward	GAGTCAACGGATTTGGTCGT
	Reverse	TTGATTTTGGAGGGATCTCG
WDR77	Forward	CTGGCTTTTTAAGGACCCCTG
	Reverse	TCTCCCCAACCCAAGTGAGG
HBV pgRNA	Forward	CTCCTCCAGCTTATAGACC
	Reverse	GTGAGTGGGCCTACAAA
cccDNA	Forward	AGCTGAGGCGGTYATCTA
	Reverse	GCCTATTGATTGGAAAGTATGT
cccDNA	Forward	CTCCCCGTCTGTGCCTTCT
	Reverse	GCCCCAAAGCCACCCAAG
DDB1	Forward	CAAAGCACGATTGTGTGCCACAATC
	Reverse	GGTCTCTCCAAGGAGTTCTACACGG

Table S7. List of antibodies used in this paper.

Antibody	Host species	Source
Flag tag	Mouse	Proteintech
Flag tag	Rabbit	Proteintech
Anti-DDDDK-tag mAb- Magnetic Agarose	Mouse	MBL
Anti-HA-tag mAb- Magnetic Agarose	Mouse	MBL
HA-tag	Mouse	MBL
HA-tag	Rabbit	MBL
PRMT5	Rabbit	Proteintech
H4R3me2s (Histone H4 symmetric di methyl R3)	Rabbit	Abcam
WDR77	Rabbit	Abcam
WDR77	Rabbit	CST
HBc	Mouse	Abcam
HBc	Mouse	Gene Tex
hALB	Rabbit	Abcam
Rme2sy	Rabbit	PTM BIO
Fluorescein (FITC)–conjugated Goat Anti-Mouse IgG(H+L)	Goat anti-mouse	Proteintech
Rhodamine (TRITC)–conjugated Goat Anti-Rabbit IgG(H+L)	Goat anti-rabbit	Proteintech
Histone H4	Rabbit	Abclonal
HBx	Mouse	Abcam
DDB1	Rabbit	Abcam
